# Supplementary material for: The effect of biomass smoke exposure on quality-of-life among Ugandan patients treated for tuberculosis: A cross-sectional analysis
Source: PLOS Glob Public Health. 2024 Feb 8;4(2):e0002892. doi: 10.1371/journal.pgph.0002892 (PMC10852290; doi:10.1371/journal.pgph.0002892)
Supplement: S1 Datakey — (PDF) [file pgph.0002892.s003.pdf]

```
#Clear existing data and graphics
rm(list=ls())
graphics.off()
#Load Hmisc library
library(Hmisc)
#Read Data
data=read.csv('TheEffectOfTheCOVID1_DATA_2022-02-17_1212.csv')
#Setting Labels

label(data$record_id)="Study ID: "
label(data$redcap_event_name)="Event Name"
label(data$study_site)="Subject Study Site:"
label(data$other_site)="Please list multiple sites separated by comma
or site not listed above."
label(data$demodate)="Date of chart abstraction."
label(data$chart_date)="Date of visit on medical chart."
label(data$tb_setting_all_d)="Please tick all events related to
tuberculosis that occurred for this subject since September, 2019"
label(data$tb_dx_time_period_d)="During what time period was the
patient diagnosed with tuberculosis"
label(data$firstname)="First name"
label(data$lastnamefather)="Complete last name"
label(data$dob)="Date of birth?"
label(data$age)="Age"
label(data$height)="Height (cm)"
label(data$weight)="Weight (kilograms)"
label(data$bmi)="BMI"
label(data$sex)="Gender"
label(data$mobilephone)="Is there a mobile phone number available for
the patient?"
label(data$personalnumcell)="What is the subjects mobile phone
number?"
label(data$personalnumhome)="What is the subjects home phone number?"
label(data$contact)="Is there a friend or relative listed who can be
contacted to reach the subject?"
label(data$relationship)="What is their relationship to the patient?"
label(data$contact_numcell)="What is the contacts phone number?"
label(data$district)="District "
label(data$county)="County"
label(data$subcounty)="Sub-county"
label(data$village)="Village"
label(data$parish)="Parish"
label(data$distance_km)="Distance between patients home parishes and
the health facilities where they seek care."
label(data$demographics_complete)="Complete?"
label(data$alive)="Is the subject still alive?"
label(data$date_death)="Approximate date of death"
label(data$reason_consent_declined)="Reason participant has declined
to consent:"
label(data$consent_read)="Consent has been read out to the
```

```
participant"
label(data$consent_obtained)="Consent has been obtained"
label(data$consent_complete)="Complete?"
label(data$tb_setting_all___1)="Please tick all events related to
tuberculosis that occurred for this subject since September, 2019
(choice=Diagnosed with tuberculosis)"
label(data$tb_setting_all___2)="Please tick all events related to
tuberculosis that occurred for this subject since September, 2019
(choice=Visited tuberculosis clinic)"
label(data$tb_setting_all___3)="Please tick all events related to
tuberculosis that occurred for this subject since September, 2019
(choice=Missed a tuberculosis clinic appointment)"
label(data$tb_setting_all___4)="Please tick all events related to
tuberculosis that occurred for this subject since September, 2019
(choice=Admitted to tuberculosis inpatient ward)"
label(data$tb_multiple)="Has the patient been diagnosed with
tuberculosis more than once?"
label(data$tb_dx_year)="Approximately what year was the patient first
diagnosed with tuberculosis?"
label(data$tb_dx_count)="How many times has the patient been diagnosed
with tuberculosis?"
label(data$tb_dx_time_period)="During what time period was the patient
diagnosed with tuberculosis"
label(data$placediagtb)="Where was the subject diagnosed with
pulmonary tuberculosis during the study period?"
label(data$tb_dx_facility_other)="Please enter the type of the other
facility:"
label(data$tb_dx_site)="What Clinic or Hospital diagnosed the patient
with tuberculosis?"
label(data$tb_dx_site_other)="Please type the name of the other clinic
or facility where the subject was diagnosed with TB"
label(data$time_to_dx)="About how long did you feel sick before you
were diagnosed with tuberculosis?"
label(data$visit_predx)="About how many times while you were sick did
you go to a clinic or hospital before you were diagnosed with
tuberculosis?"
label(data$tb_dx_barrier___1)="Please select anything below that you
feel may have delayed getting your diagnosis of TB or made getting
diagnosed difficult ? (choice=Transportation to clinics or doctors)"
label(data$tb_dx_barrier___2)="Please select anything below that you
feel may have delayed getting your diagnosis of TB or made getting
diagnosed difficult ? (choice=Distance to clinics or doctors)"
label(data$tb_dx_barrier___3)="Please select anything below that you
feel may have delayed getting your diagnosis of TB or made getting
diagnosed difficult ? (choice=The cost of clinics or doctors)"
label(data$tb_dx_barrier___4)="Please select anything below that you
feel may have delayed getting your diagnosis of TB or made getting
diagnosed difficult ? (choice=Lack of doctors or clinics who you
thought could see you)"
label(data$tb_dx_barrier___5)="Please select anything below that you
```

```
feel may have delayed getting your diagnosis of TB or made getting
diagnosed difficult ? (choice=Busy with work)"
label(data$tb_dx_barrier___6)="Please select anything below that you
feel may have delayed getting your diagnosis of TB or made getting
diagnosed difficult ? (choice=Busy with family obligations (child
care, caring for a mother or father etc.))"
label(data$tb_dx_barrier___7)="Please select anything below that you
feel may have delayed getting your diagnosis of TB or made getting
diagnosed difficult ? (choice=Your physical condition or sickness)"
label(data$tb_dx_barrier___8)="Please select anything below that you
feel may have delayed getting your diagnosis of TB or made getting
diagnosed difficult ? (choice=No family or friends to help you get to
the doctor or clinic)"
label(data$tb_dx_barrier___9)="Please select anything below that you
feel may have delayed getting your diagnosis of TB or made getting
diagnosed difficult ? (choice=Not sure that a doctor or clinic would
help)"
label(data$tb_dx_barrier___10)="Please select anything below that you
feel may have delayed getting your diagnosis of TB or made getting
diagnosed difficult ? (choice=Concern about going to see a doctor or
go to a clinic)"
label(data$tb_dx_barrier___11)="Please select anything below that you
feel may have delayed getting your diagnosis of TB or made getting
diagnosed difficult ? (choice=Worry about getting another disease if
you went to a doctor or clinic)"
label(data$tb_dx_barrier___12)="Please select anything below that you
feel may have delayed getting your diagnosis of TB or made getting
diagnosed difficult ? (choice=Tried other medicine such as traditional
medicine or herbs first)"
label(data$tb_dx_barrier___13)="Please select anything below that you
feel may have delayed getting your diagnosis of TB or made getting
diagnosed difficult ? (choice=There wasnt anything that made it hard
to get diagnosed with tuberculosis)"
label(data$tb_dx_barrier___14)="Please select anything below that you
feel may have delayed getting your diagnosis of TB or made getting
diagnosed difficult ? (choice=Other reason not related to above)"
label(data$dx_barrier_other)="Please list other things that made
getting diagnosed with tuberculosis difficult?"
label(data$tb_clinic_timeperiod___1)="During what time period did the
patient attend TB clinic, please check all that apply. (choice=Prior
to March 15th, 2020)"
label(data$tb_clinic_timeperiod___2)="During what time period did the
patient attend TB clinic, please check all that apply. (choice=March
15th-June, 2020)"
label(data$tb_clinic_timeperiod___3)="During what time period did the
patient attend TB clinic, please check all that apply. (choice=July,
2020 or after)"
label(data$tb_clinic_timeperiod___4)="During what time period did the
patient attend TB clinic, please check all that apply. (choice=None of
these time periods)"
```

```
label(data$tp_clnic_complete)="During what time period did the subject
complete their TB treatment?"
label(data$missedclinic_lockdown)="Did this patient miss clinic during
lockdown (March 15th, 2020-June, 2020 "
label(data$tb_clinic_other)="Has the patient attended another TB
clinic aside from the clinic recorded on the demographic form?"
label(data$tb_clinic_other_name)="Please list the other clinic the
subject attended here"
label(data$tb_clinic_satisfaction)="Do you feel that going to
tuberculosis clinic helped you get better?"
label(data$tb_clinic_satisfaction_2)="Do you feel that the doctors and
nurses at the tuberculosis clinic took enough time taking care for
you?"
label(data$tb_missed_clinic_date___1)="In what time period did the
patient miss a tuberculosis clinic appointment? (choose all that
apply) (choice=Prior to March 15th, 2020)"
label(data$tb_missed_clinic_date___2)="In what time period did the
patient miss a tuberculosis clinic appointment? (choose all that
apply) (choice=March 15th - June 2020)"
label(data$tb_missed_clinic_date___3)="In what time period did the
patient miss a tuberculosis clinic appointment? (choose all that
apply) (choice=After June, 2020)"
label(data$tb_missedclinic_other)="Did the patient miss any clinics at
a clinic not recorded on the initial intake form?"
label(data$tb_missedclinic_other_name)="What is the name of the TB
clinic where you missed this appointment?"
label(data$missed_reason___1)="What were some of the reasons that you
missed clinic? (choice=Transportation to clinics or doctors)"
label(data$missed_reason___2)="What were some of the reasons that you
missed clinic? (choice=Distance to clinics or doctors)"
label(data$missed_reason___3)="What were some of the reasons that you
missed clinic? (choice=The cost of clinics or doctors)"
label(data$missed_reason___4)="What were some of the reasons that you
missed clinic? (choice=Lack of doctors or clinics who you thought
could see you)"
label(data$missed_reason___5)="What were some of the reasons that you
missed clinic? (choice=Busy with work)"
label(data$missed_reason___6)="What were some of the reasons that you
missed clinic? (choice=Busy with family obligations (child care,
caring for a mother or father etc.))"
label(data$missed_reason___7)="What were some of the reasons that you
missed clinic? (choice=Your physical condition or sickness)"
label(data$missed_reason___8)="What were some of the reasons that you
missed clinic? (choice=No family or friends to help you get to the
doctor or clinic)"
label(data$missed_reason___9)="What were some of the reasons that you
missed clinic? (choice=Not sure that a doctor or clinic would help)"
label(data$missed_reason___10)="What were some of the reasons that you
missed clinic? (choice=Concern about going to see a doctor or go to a
clinic)"
```

```
label(data$missed_reason___11)="What were some of the reasons that you
missed clinic? (choice=Worry about getting another disease if you went
to a doctor or clinic)"
label(data$missed_reason___12)="What were some of the reasons that you
missed clinic? (choice=Wanted to try other medicine such as
traditional medicine or herbs first)"
label(data$missed_reason___13)="What were some of the reasons that you
missed clinic? (choice=There wasnt anything that made you miss
clinic)"
label(data$missed_reason___14)="What were some of the reasons that you
missed clinic? (choice=Other reasons not listed above)"
label(data$missed_other)="Please specify any other reasons you missed
clinic?"
label(data$tb_admitted_date)="In what time period was the patient
admitted to the tuberculosis ward? "
label(data$tb_ward_name)="What is the name of the tuberculosis ward
the subject was admitted to?"
label(data$time_to_ward)="About how long did you feel sick before you
were admitted to the TB hospital or ward?"
label(data$tb_ward_reason___1)="Please select anything that you felt
delayed you getting admitted to the TB ward or hospital??
(choice=Transportation)"
label(data$tb_ward_reason___2)="Please select anything that you felt
delayed you getting admitted to the TB ward or hospital??
(choice=Distance to TB ward or doctors)"
label(data$tb_ward_reason___3)="Please select anything that you felt
delayed you getting admitted to the TB ward or hospital?? (choice=The
cost of doctors or the hospital ward)"
label(data$tb_ward_reason___4)="Please select anything that you felt
delayed you getting admitted to the TB ward or hospital?? (choice=Lack
of hospitals who you thought could see or care for you)"
label(data$tb_ward_reason___5)="Please select anything that you felt
delayed you getting admitted to the TB ward or hospital?? (choice=Busy
with work)"
label(data$tb_ward_reason___6)="Please select anything that you felt
delayed you getting admitted to the TB ward or hospital?? (choice=Busy
with family obligations (child care, caring for a mother or father
etc.))"
label(data$tb_ward_reason___7)="Please select anything that you felt
delayed you getting admitted to the TB ward or hospital?? (choice=Your
physical condition or sickness)"
label(data$tb_ward_reason___8)="Please select anything that you felt
delayed you getting admitted to the TB ward or hospital?? (choice=No
family or friends to help you get to the doctor or TB ward)"
label(data$tb_ward_reason___9)="Please select anything that you felt
delayed you getting admitted to the TB ward or hospital?? (choice=Not
sure that a doctor or TB ward would help)"
label(data$tb_ward_reason___10)="Please select anything that you felt
delayed you getting admitted to the TB ward or hospital??
(choice=Concern about being admitted to the TB ward)"
```

```
label(data$tb_ward_reason___11)="Please select anything that you felt
delayed you getting admitted to the TB ward or hospital??
(choice=Worry about getting another disease if you were admitted to
the TB ward or hospital)"
label(data$tb_ward_reason___12)="Please select anything that you felt
delayed you getting admitted to the TB ward or hospital??
(choice=Wanted to try other medicine such as traditional medicine or
herbs first)"
label(data$tb_ward_reason___13)="Please select anything that you felt
delayed you getting admitted to the TB ward or hospital??
(choice=There wasnt anything that delayed you being admitted to the TB
ward)"
label(data$tb_ward_reason___14)="Please select anything that you felt
delayed you getting admitted to the TB ward or hospital??
(choice=Other reasons not listed above)"
label(data$tb_ward_other)="Please specify any other reasons you
delayed going to the TB ward?"
label(data$tb_ward_perception)="Do you feel that being admitted to the
TB ward or hospital made you better?"
label(data$tb_ward_perception_2)="Do you feel that the doctors and
nurses in the TB ward or hospital had enought time to care for you?"
label(data$tbtreatment)="Did the subject complete treatment for your
pulmonary tuberculosis?"
label(data$tb_med___1)="What medications have you taken or are you
taking for tuberculosis (check all that apply)? (choice=2RHZE/4HR
(daily ethambutol (EMB), isoniazid (INH), rifampicin (RMP) and
pyrazinamide (PZA) for 2 months followed by EMB and INH for 4
months))"
label(data$tb_med___2)="What medications have you taken or are you
taking for tuberculosis (check all that apply)? (choice=2RHZE/10RH
(daily ethambutol (EMB), isoniazid (INH), rifampicin (RMP) and
pyrazinamide (PZA) for 2 months followed by EMB and INH for 10
months))"
label(data$tb_med___3)="What medications have you taken or are you
taking for tuberculosis (check all that apply)? (choice=No
medications)"
label(data$tb_med___4)="What medications have you taken or are you
taking for tuberculosis (check all that apply)? (choice=Other)"
label(data$tb_med_other)="What are the names of the other medications
you have taken or are taking for tuberculosis?"
label(data$tb_complete)="Was the subject able to complete this form?"
label(data$tb_complete_reason___1)="Why was the subject unable to
complete this form? (choice=Phone time was limited or phone cut off)"
label(data$tb_complete_reason___2)="Why was the subject unable to
complete this form? (choice=Busy and asked for call back)"
label(data$tb_complete_reason___3)="Why was the subject unable to
complete this form? (choice=Busy and asked to not be called back)"
label(data$tb_complete_reason___4)="Why was the subject unable to
complete this form? (choice=Refused to answer further questions and
asked to not be called back)"
```

```
label(data$tb_complete_reason___5)="Why was the subject unable to
complete this form? (choice=Other)"
label(data$tuberculosis_questionnaire_complete)="Complete?"
label(data$pmhx___1)="Please check all conditions that the subject has
in addition to tuberculosis (choice=HTN)"
label(data$pmhx___2)="Please check all conditions that the subject has
in addition to tuberculosis (choice=CAD)"
label(data$pmhx___3)="Please check all conditions that the subject has
in addition to tuberculosis (choice=Stroke)"
label(data$pmhx___4)="Please check all conditions that the subject has
in addition to tuberculosis (choice=Heart Failure)"
label(data$pmhx___5)="Please check all conditions that the subject has
in addition to tuberculosis (choice=Other Heart Disease)"
label(data$pmhx___6)="Please check all conditions that the subject has
in addition to tuberculosis (choice=DM)"
label(data$pmhx___7)="Please check all conditions that the subject has
in addition to tuberculosis (choice=Asthma)"
label(data$pmhx___8)="Please check all conditions that the subject has
in addition to tuberculosis (choice=CB)"
label(data$pmhx___9)="Please check all conditions that the subject has
in addition to tuberculosis (choice=Emphysema)"
label(data$pmhx___10)="Please check all conditions that the subject
has in addition to tuberculosis (choice=COPD)"
label(data$pmhx___11)="Please check all conditions that the subject
has in addition to tuberculosis (choice=Cancer)"
label(data$pmhx___12)="Please check all conditions that the subject
has in addition to tuberculosis (choice=HIV/AIDS)"
label(data$pmhx___13)="Please check all conditions that the subject
has in addition to tuberculosis (choice=No Medical History)"
label(data$pmhx___14)="Please check all conditions that the subject
has in addition to tuberculosis (choice=Other)"
label(data$med_hx_other)="Please list other medical conditions not
listed above"
label(data$regularmeds___1)="Do the clinic notes state that the
subject has taken any of the following medications? Select all.
(choice=Inhaled steroids)"
label(data$regularmeds___2)="Do the clinic notes state that the
subject has taken any of the following medications? Select all.
(choice=Short acting beta-agonists (in any form))"
label(data$regularmeds___3)="Do the clinic notes state that the
subject has taken any of the following medications? Select all.
(choice=Short acting anti-muscarinic bronchodilator (eg. Ipratropium
bromide))"
label(data$regularmeds___4)="Do the clinic notes state that the
subject has taken any of the following medications? Select all.
(choice=Long acting beta-agonists (in any form))"
label(data$regularmeds___5)="Do the clinic notes state that the
subject has taken any of the following medications? Select all.
(choice=Long acting anti-muscarinics bronchodilator (in any form eg.
Tiotropium))"
```

```
label(data$regularmeds___6)="Do the clinic notes state that the
subject has taken any of the following medications? Select all.
(choice=Xanthines (theophylline))"
label(data$regularmeds___7)="Do the clinic notes state that the
subject has taken any of the following medications? Select all.
(choice=Non-inhaled steroids (in any form, e.g. IV, depot injections,
tablet))"
label(data$other_med)="Does the patient take any other medications?"
label(data$other_med_type)="Please list any other medications the
subject takes, separated by commas"
label(data$chestinfections)="How many times prior to TB care
(diagnosis, clinic visit or ward admission) did the subject receive
non-TB antibiotics for a chest infection?"
label(data$exp_cig)="Has the subject ever smoked cigarettes?"
label(data$exp_cig_yrstart)="What year did the patient start smoking
cigarettes?"
label(data$exp_cig_current)="Does the patient still smoke cigarettes?"
label(data$exp_cig_tb)="Did the subject smoke cigarettes at the time
of TB diagnosis?"
label(data$exp_cig_yrs)="How many years total did the subject smoke
cigarettes?"
label(data$exp_cig_day)="On average, how many cigarettes does the
patient smoke per day?"
label(data$exp_cig_pkyr)="Total Pack Years"
label(data$exp_smoke)="Has the subject ever smoked anything else?"
label(data$exp_pipe)="Has the subject ever smoked a pipe?"
label(data$exp_pipe_now)="Does the subject currently smoke a pipe?"
label(data$exp_pipe_day)="Number of times a day the subject smokes a
pipe?"
label(data$exp_pipeyr)="How many years has the subject smoked a pipe?"
label(data$exp_cigar)="Has the subject ever smoked cigars or rolled
leaves to smoke?"
label(data$exp_cigar_now)="Does the subject currently smoke cigars or
roll leaves to smoke?"
label(data$exp_cigar_wk)="How many times a week does the subject smoke
cigars or roll leaves to smoke?"
label(data$exp_cigar_yr)="How many years has the subject smoked cigars
or rolled leaves to smoke?"
label(data$exp_shisha)="Has the subject ever smoked shisha or a water-
pipe?"
label(data$wami_sanitation)="What kind of toilet facility do members
of your household usually use? "
label(data$wami_water)="What is the main  source of  drinking water
for members of your household? "
label(data$wami_asset_cookroom)="Do you have a separate room for
cookings outside of the main living area?"
label(data$wami_asset_multiple___1)="Does any member of your household
have any of the following: (choice=Bank Account)"
label(data$wami_asset_multiple___2)="Does any member of your household
have any of the following: (choice=Mattress)"
```

```
label(data$wami_asset_multiple___3)="Does any member of your household have any of the following: (choice=Refrigerator)"
label(data$wami_asset_multiple___4)="Does any member of your household have any of the following: (choice=Television)"
label(data$wami_asset_multiple___5)="Does any member of your household have any of the following: (choice=Table)"
label(data$wami_asset_multiple___6)="Does any member of your household have any of the following: (choice=Chair or bench)"
label(data$wami_asset_household)="How many total people, including the patient, usually sleep (live) in the household?"
label(data$wami_income)="What is the average income for the entire household, including the contributions of all members?"
label(data$incometime)="Per:"
label(data$underfive)="How many children under the age of five live in the household?"
label(data$employment___1)="What was the subjects occupational status in the last 6 months? (choice=Employed in agricultural/farming/forestry/fishing)"
label(data$employment___2)="What was the subjects occupational status in the last 6 months? (choice=Employed in formal government/public sector)"
label(data$employment___3)="What was the subjects occupational status in the last 6 months? (choice=Employed in formal private sector)"
label(data$employment___4)="What was the subjects occupational status in the last 6 months? (choice=Employed in the informal sector)"
label(data$employment___5)="What was the subjects occupational status in the last 6 months? (choice=Unpaid domestic tasks and/or care responsibilities (e.g. housewife))"
label(data$employment___6)="What was the subjects occupational status in the last 6 months? (choice=Student)"
label(data$employment___7)="What was the subjects occupational status in the last 6 months? (choice=Disabled to work due to any health issue)"
label(data$employment___8)="What was the subjects occupational status in the last 6 months? (choice=Retired/Pensioner)"
label(data$employment___9)="What was the subjects occupational status in the last 6 months? (choice=Unemployed)"
label(data$employment___10)="What was the subjects occupational status in the last 6 months? (choice=Other)"
label(data$employment____99)="What was the subjects occupational status in the last 6 months? (choice=No answer)"
label(data$hohwork)="Does the head of the household currently work for income or for food for your household?"
label(data$houseyears)="For how many years has the subject lived in their current house?"
label(data$marital_status)="Is the subject currently married?"
label(data$cooking)="Is the patient responsible for cooking for their household? "
label(data$stovetype___1)="What type of cooking stoves are used in the house? Select all that are in use.   (choice=Open fire)"
```

```
label(data$stovetype___2)="What type of cooking stoves are used in the house? Select all that are in use.   (choice=Biomass stove with chimney)"
label(data$stovetype___3)="What type of cooking stoves are used in the house? Select all that are in use.   (choice=Biomass stove without chimney)"
label(data$stovetype___4)="What type of cooking stoves are used in the house? Select all that are in use.   (choice=Liquified petroleum gas (LPG))"
label(data$stovetype___5)="What type of cooking stoves are used in the house? Select all that are in use.   (choice=Natural Gas)"
label(data$stovetype___6)="What type of cooking stoves are used in the house? Select all that are in use.   (choice=Kerosene)"
label(data$stovetype___7)="What type of cooking stoves are used in the house? Select all that are in use.   (choice=Electricity)"
label(data$stovetype___8)="What type of cooking stoves are used in the house? Select all that are in use.   (choice=Other)"
label(data$cookingarea)="Is the cooking done inside the house, outside the house or both?"
label(data$separatekitchen)="Is there a separate room for cooking outside of the main living area?"
label(data$fuelsource)="What is the primary source of fuel used for cooking in the household?"
label(data$bioyears)="For how many years has the subject been cooking with biomass fuels?"
label(data$biomass)="Do they burn biomass for cooking  daily? "
label(data$biohours)="How many hours per day does the subject spend cooking with biomass fuels?"
label(data$biofuels___1)="What biomass fuels does the subject burn? (select all that you use) (choice=Wood)"
label(data$biofuels___2)="What biomass fuels does the subject burn? (select all that you use) (choice=Charcoal)"
label(data$biofuels___3)="What biomass fuels does the subject burn? (select all that you use) (choice=Dung/Animal Waste)"
label(data$biofuels___4)="What biomass fuels does the subject burn? (select all that you use) (choice=Agricultural/Crop Waste)"
label(data$biofuels___5)="What biomass fuels does the subject burn? (select all that you use) (choice=None of these)"
label(data$pastbiomass)="Has the subject ever lived in a household that burned biomass fuel (wood, charcoal, animal or crop waste...) for cooking?"
label(data$pastbiomassyears)="For how many years did they live in a household that burned biomass fuel for cooking?"
label(data$idi)="Would you be willing to take part in a in-person interview which will last approximately 45 minutes related to the care you received for tuberculosis?  (Transportation to the interview site and refreshments will be provided)"
label(data$tb_complete_2)="Was the subject able to complete this form?"
label(data$tb_complete_reason_2___1)="Why was the subject unable to
```

```
complete this form? (choice=Phone time was limited or phone cut off)"
label(data$tb_complete_reason_2___2)="Why was the subject unable to
complete this form? (choice=Busy and asked for call back)"
label(data$tb_complete_reason_2___3)="Why was the subject unable to
complete this form? (choice=Busy and asked to not be called back)"
label(data$tb_complete_reason_2___4)="Why was the subject unable to
complete this form? (choice=Refused to answer further questions and
asked to not be called back)"
label(data$tb_complete_reason_2___5)="Why was the subject unable to
complete this form? (choice=Other)"
label(data$phone_questionnaire_general_complete)="Complete?"
label(data$everwheeze)="Have you ever had wheezing (whistles, squeaks,
rattles, or buzzes) in your chest?"
label(data$recentwheeze)="Have you had wheezing (whistles, squeaks,
rattles, or buzzes) in your chest in the last 12 months?"
label(data$seekcare)="When you have wheezing in the chest or shortness
of breath,  where do you seek care?"
label(data$cough)="Do you have cough with phlegm on most days of the
week?"
label(data$cbronch)="Have you brought up phlegm with a cough that
occurs most days and that lasts for at least 3 months, 2 years in a
row?"
label(data$hospitalized)="In the past 12 months, have you ever been
hospitalized due to your respiratory problems? "
label(data$impeded)="In the past 12 months, have you had to miss work
or have your daily activities been impeded because of your respiratory
problems? "
label(data$cap5)="In the past 12 months, how many times did you miss
work, school, or other activities due to a cold, bronchitis, or
pneumonia?"
label(data$eq1)="Mobility (Okutambula) "
label(data$eq2)="Self-Care (Okwefaako)"
label(data$eq3)="Usual Activities (e.g. work, study, housework, family
or leisure activities) (Ebintu ebikolebwa bulijjo (Gamba nga, okukola,
okusoma, emirimu gyawaka, ebyomumaka oba ebikolebwa mu biseera
ebyegandaalo))"
label(data$eq4)="Pain / Discomfort (Obulumi/Obutewulira bulungi)"
label(data$eq5)="Anxiety / Depression (Okwelarikirira oba enyiike)"
label(data$eq6)="Response"
label(data$tb_complete_3)="Was the subject able to complete this
form?"
label(data$tb_complete_reason_3___1)="Why was the subject unable to
complete this form? (choice=Phone time was limited or phone cut off)"
label(data$tb_complete_reason_3___2)="Why was the subject unable to
complete this form? (choice=Busy and asked for call back)"
label(data$tb_complete_reason_3___3)="Why was the subject unable to
complete this form? (choice=Busy and asked to not be called back)"
label(data$tb_complete_reason_3___4)="Why was the subject unable to
complete this form? (choice=Refused to answer further questions and
asked to not be called back)"
```

```
label(data$tb_complete_reason_3___5)="Why was the subject unable to complete this form? (choice=Other)"
label(data$respiratory_and_eq5d3l_complete)="Complete?"
label(data$phq1)="Little interest or pleasure in doing things"
label(data$phq2)="Feeling down, depressed, or hopeless"
label(data$phq3)="Trouble falling or staying asleep, or sleeping too much"
label(data$phq4)="Feeling tired or having little energy"
label(data$phq5)="Poor appetite or overeating"
label(data$phq6)="Feeling bad about yourself - or that you are a failure or have let yourself or your family down "
label(data$phq7)="Trouble concentrating on things, such as reading the newspaper or watching television"
label(data$phq8)="Moving or speaking so slowly that other people could have noticed. Or the opposite - being so fidgety or restless that you have been moving around a lot more than usual"
label(data$phq9)="Thoughts that you would be better off dead, or of hurting yourself in some way"
label(data$phq10)="If you checked off any problems, how difficult have these problems made it for you to do your work, take care of things at home, or get along with other people?"
label(data$tb_complete_4)="Was the subject able to complete this form?"
label(data$tb_complete_reason_4___1)="Why was the subject unable to complete this form? (choice=Phone time was limited or phone cut off)"
label(data$tb_complete_reason_4___2)="Why was the subject unable to complete this form? (choice=Busy and asked for call back)"
label(data$tb_complete_reason_4___3)="Why was the subject unable to complete this form? (choice=Busy and asked to not be called back)"
label(data$tb_complete_reason_4___4)="Why was the subject unable to complete this form? (choice=Refused to answer further questions and asked to not be called back)"
label(data$tb_complete_reason_4___5)="Why was the subject unable to complete this form? (choice=Other)"
label(data$phq9_complete)="Complete?"
label(data$sgrq_current_health)="Before completing the rest of the questionnaire: Please tick in one circle to show how you describe your current health:   Oyinza kunnyonyola otya embeera yobulamu mukaseera kano?"
label(data$sgrq_1)="1. Over the past 3 months, I have coughed: / Mu myezi essatu (3) eziyise nkolodde:"
label(data$sgrq_2)="2. Over the past 3 months, I have brought up phlegm (sputum):  / Mu myezi essatu (3) eziyise mbadde nvaamu ekikolondolwa:"
label(data$sgrq_3)="3. Over the past 3 months, I have had shortness of breath: / Mu myezi essatu (3) eziyise mbadde nziyira:"
label(data$sgrq_4)="4. Over the past 3 months, I have had attacks of wheezing: / Mu myezi essatu (3) eziyise ekifuba kibadde kikaaba:"
label(data$sgrq_5)="5. During the past 3 months how many severe or very unpleasant attacks of chest trouble have you had? / Mu myezi
```

```
essatu (3) eziyise ofunye obuzibu mu kussa emirundi emeka?  Please
tick one:"
label(data$sgrq_6)="6. How long did the worst attack of chest trouble
last? / Obuzibu mu kussa bwamala bbanga ki?  Please tick one:"
label(data$sgrq_7)="7. Over the past 3 months, in an average week, how
many good days (with little chest trouble) have you had?  Mu myezi
essatu (3) eziyise, mu buli wiiki obadde ofuna ennaku ennungi meka?
Please tick one:"
label(data$sgrq_8)="8. If you have a wheeze, is it worse in the
morning? Bwoba ofunye ekifuba kibadde kikaaba, kisinga kubeerawo mu
budde bwakumakya?  Please tick one:"
label(data$sgrq_sect_1_1)="How would you describe your chest
condition? / Oyinza kunnyonyola otya embeera yekifuba kyo?  Please
tick one:"
label(data$sgrq_sect_1_2)="If you have ever had paid employment. /
Bwoba wali ofunye omumulimu nga bakusasula:  Please tick one:"
label(data$sgrq_sect_2_1)="Sitting or lying still / Okutuula oba
okugalamira"
label(data$sgrq_sect_2_2)="Getting washed or dressed / Okwooza o
okwambala"
label(data$sgrq_sect_2_3)="Walking around the home / Okutambula ewaka"
label(data$sgrq_sect_2_4)="Walking outside on the level / Okutambula
ku museetwe/awatelevu"
label(data$sgrq_sect_2_5)="Walking up a flight of stairs / Okulinnya
amadaala"
label(data$sgrq_sect_2_6)="Walking up hills / Okulinnya akasozi"
label(data$sgrq_sect_2_7)="Playing sports or games / Okuzanya
emizannyo"
label(data$sgrq_sect_3_1)="My cough hurts / Okukolola kundetera
obulumi"
label(data$sgrq_sect_3_2)="My cough makes me tired / Okukolola
kunkooya"
label(data$sgrq_sect_3_3)="I am breathless when I talk / Nziyira
bwemba nga njogera"
label(data$sgrq_sect_3_4)="I am breathless when I bend over / Nziyira
bweneeweta"
label(data$sgrq_sect_3_5)="My cough or breathing disturbs my sleep /
Okukolola oba okuziyira kuntawanya nga nneebase"
label(data$sgrq_sect_3_6)="I get exhausted easily / Nkoowa mangu"
label(data$sgrq_sect_4_1)="My cough or breathing is embarrassing in
public / Okukolola oba okuziyira kunswaza mu bantu"
label(data$sgrq_sect_4_2)="My chest trouble is a nuisance to my
family, friends or neighbours / Obuzibu mukusa kwange bwafuuka
ekyomutawaana mu famile, mikwano ne balirwana"
label(data$sgrq_sect_4_3)="I get afraid or panic when I cannot get my
breath / Ntya bwemba sisobola kussa bulungi"
label(data$sgrq_sect_4_4)="I feel that I am not in control of my chest
problem / Mpulira nga sisobola kufuga obuzibu mukusa kyange"
label(data$sgrq_sect_4_5)="I do not expect my chest to get any
better / Sirowooza nga obuzibu mukusa kyange kinaatereera"
```

```
label(data$sgrq_sect_4_6)="I have become frail or an invalid because
of my chest / Nfuuse atakyasaanira olwekifuba kyange"
label(data$sgrq_sect_4_7)="Exercise is not safe for me / Duyiro
wabulabe ku bulamu bwange"
label(data$sgrq_sect_4_8)="Everything seems too much of an effort /
Nzibuwalirwa okukola buli kintu"
label(data$sgrq_sec_5_0)="I use medications for my chest or
breathing? / Olina edagala lyobuzibu mukusa lyoliko?"
label(data$sgrq_sect_5_1)="My medication does not help me very much /
Eddagala lyange telinnyambye nyo"
label(data$sgrq_sect_5_2)="I get embarrassed using my medication in
public / Nswala okukozesa eddagala lyange mubantu"
label(data$sgrq_sect_5_3)="I have unpleasant side effects from my
medication / Nfunye obuzibu obuvudde ku dagala lyange"
label(data$sgrq_sect_5_4)="My medication interferes with my life a
lot / Eddagala lyange likosa nyo obulamu bwange"
label(data$sgrq_sect_6_1)="I take a long time to get washed or
dressed / Kintwalina ekiseera kiwanvu okunaaba oba okwambala"
label(data$sgrq_sect_6_2)="I cannot take a bath or shower, or I take a
long time / Sisobola kunaaba oba ntwala ebbanga ddene"
label(data$sgrq_sect_6_3)="I walk slower than other people, or I stop
for rests / Ntambula mpola nyo okunsinga abantu abalala/nsooka ne
mpummulamu"
label(data$sgrq_sect_6_4)="Jobs such as housework take a long time, or
I have to stop for rests / Emirimu nga ejawaka ngintwalira ebbanga
ddene oba mbeera nina okuwummulamu"
label(data$sgrq_sect_6_5)="If I walk up one flight of stairs, I have
to go slowly or stop / Bweninya amaddaala nina okuwummulamu oba
nenkoma awo radio"
label(data$sgrq_sect_6_6)="If I hurry or walk fast, I have to stop or
slow down / Bwemba nyanguwa nina okuwummulamu oba okukendeeza ku
supiidi"
label(data$sgrq_sect_6_7)="My breathing makes it difficult to do
things such as walk up hills, carrying things up stairs, light
gardening such as weeding, dance, play bowls or play golf / Okussa
kwange kumpa obuzibu mu kukola emirimu egimu okugeza okulinya akasozi,
okwetikka, obulimu obutono, okuzina"
label(data$sgrq_sect_6_8)="My breathing makes it difficult to do
things such as carry heavy loads, dig the garden or shovel snow, jog
or walk at 5 miles per hour, play tennis or swim / Okussa kwange kumpa
obuzibu mu kukola emirimu egimu okugeza okwetikka ebizito, okudduka,
okulima"
label(data$sgrq_sect_6_9)="My breathing makes it difficult to do
things such as very heavy manual work, run, cycle, swim fast or play
competitive sports / Okussa kwange kumpa obuzibu mu kukola emirimu
egimu okugeza emirimu egyamanyi ennyo, okudduka oba okuzannya"
label(data$sgrq_sect_7_1)="I cannot play sports or games / Sisobola
kwetaba mu bwemizannyo"
label(data$sgrq_sect_7_2)="I cannot go out for entertainment or
recreation / Sisobola kugenda mu binyumu"
```

```
label(data$sgrq_sect_7_3)="I cannot go out of the house to do the
shopping / Sisobola kufuluma waka kugenda mu katale"
label(data$sgrq_sect_7_4)="I cannot do housework / Sisobola kukola
mirimu gyawaka"
label(data$sgrq_sect_7_5)="I cannot move far from my bed or chair /
Sisobola kugenda wala okuva kukitanda kyange oba entebbe yange"
label(data$sgrq_trouble)="Please write in any other important
activities that your chest trouble may stop you doing:"
label(data$sgrq_affects)="Now would you tick in the circle (one only)
which you think best describes how your chest affects you: / Kiliwa
kubino ekisinga okunyonyola obulungi engeli ekilfubakyo
gyekikukosamu?"
label(data$tb_complete_5)="Was the subject able to complete this
form?"
label(data$tb_complete_reason_5___1)="Why was the subject unable to
complete this form? (choice=Phone time was limited or phone cut off)"
label(data$tb_complete_reason_5___2)="Why was the subject unable to
complete this form? (choice=Busy and asked for call back)"
label(data$tb_complete_reason_5___3)="Why was the subject unable to
complete this form? (choice=Busy and asked to not be called back)"
label(data$tb_complete_reason_5___4)="Why was the subject unable to
complete this form? (choice=Refused to answer further questions and
asked to not be called back)"
label(data$tb_complete_reason_5___5)="Why was the subject unable to
complete this form? (choice=Other)"
label(data$appendix1_st_georges_respiratory_questionnaire_sgr_complete
)="Complete?"
#Setting Units

units(data$height)="cm"
units(data$weight)="kilograms"
units(data$bmi)="kilograms"

#Setting Factors(will create new variable for factors)
data$redcap_event_name.factor =
factor(data$redcap_event_name,levels=c("screening_data_arm_1","study_d
ata_arm_1"))
data$study_site.factor =
factor(data$study_site,levels=c("1","2","3","4","5","6","7"))
data$tb_setting_all_d.factor =
factor(data$tb_setting_all_d,levels=c("1","2","3","4"))
data$tb_dx_time_period_d.factor =
factor(data$tb_dx_time_period_d,levels=c("1","2","3","4"))
data$sex.factor = factor(data$sex,levels=c("0","1"))
data$mobilephone.factor = factor(data$mobilephone,levels=c("1","0"))
data$contact.factor = factor(data$contact,levels=c("1","0"))
data$relationship.factor =
factor(data$relationship,levels=c("1","2","3","4","5","6","7"))
data$demographics_complete.factor =
factor(data$demographics_complete,levels=c("0","1","2"))
```

```
data$alive.factor = factor(data$alive,levels=c("1","0"))
data$consent_read.factor = factor(data$consent_read,levels=c("1","0"))
data$consent_obtained.factor =
factor(data$consent_obtained,levels=c("1","0"))
data$consent_complete.factor =
factor(data$consent_complete,levels=c("0","1","2"))
data$tb_setting_all___1.factor =
factor(data$tb_setting_all___1,levels=c("0","1"))
data$tb_setting_all___2.factor =
factor(data$tb_setting_all___2,levels=c("0","1"))
data$tb_setting_all___3.factor =
factor(data$tb_setting_all___3,levels=c("0","1"))
data$tb_setting_all___4.factor =
factor(data$tb_setting_all___4,levels=c("0","1"))
data$tb_multiple.factor = factor(data$tb_multiple,levels=c("1","0"))
data$tb_dx_time_period.factor =
factor(data$tb_dx_time_period,levels=c("1","2","3"))
data$placediagtb.factor =
factor(data$placediagtb,levels=c("1","2","3","4","5","6"))
data$tb_dx_site.factor =
factor(data$tb_dx_site,levels=c("1","2","3","4","5","6","7"))
data$time_to_dx.factor =
factor(data$time_to_dx,levels=c("1","2","3","4","5"))
data$tb_dx_barrier___1.factor =
factor(data$tb_dx_barrier___1,levels=c("0","1"))
data$tb_dx_barrier___2.factor =
factor(data$tb_dx_barrier___2,levels=c("0","1"))
data$tb_dx_barrier___3.factor =
factor(data$tb_dx_barrier___3,levels=c("0","1"))
data$tb_dx_barrier___4.factor =
factor(data$tb_dx_barrier___4,levels=c("0","1"))
data$tb_dx_barrier___5.factor =
factor(data$tb_dx_barrier___5,levels=c("0","1"))
data$tb_dx_barrier___6.factor =
factor(data$tb_dx_barrier___6,levels=c("0","1"))
data$tb_dx_barrier___7.factor =
factor(data$tb_dx_barrier___7,levels=c("0","1"))
data$tb_dx_barrier___8.factor =
factor(data$tb_dx_barrier___8,levels=c("0","1"))
data$tb_dx_barrier___9.factor =
factor(data$tb_dx_barrier___9,levels=c("0","1"))
data$tb_dx_barrier___10.factor =
factor(data$tb_dx_barrier___10,levels=c("0","1"))
data$tb_dx_barrier___11.factor =
factor(data$tb_dx_barrier___11,levels=c("0","1"))
data$tb_dx_barrier___12.factor =
factor(data$tb_dx_barrier___12,levels=c("0","1"))
data$tb_dx_barrier___13.factor =
factor(data$tb_dx_barrier___13,levels=c("0","1"))
data$tb_dx_barrier___14.factor =
```

```
factor(data$tb_dx_barrier___14,levels=c("0","1"))
data$tb_clinic_timeperiod___1.factor =
factor(data$tb_clinic_timeperiod___1,levels=c("0","1"))
data$tb_clinic_timeperiod___2.factor =
factor(data$tb_clinic_timeperiod___2,levels=c("0","1"))
data$tb_clinic_timeperiod___3.factor =
factor(data$tb_clinic_timeperiod___3,levels=c("0","1"))
data$tb_clinic_timeperiod___4.factor =
factor(data$tb_clinic_timeperiod___4,levels=c("0","1"))
data$tp_clnic_complete.factor =
factor(data$tp_clnic_complete,levels=c("1","2","3","4"))
data$missedclinic_lockdown.factor =
factor(data$missedclinic_lockdown,levels=c("1","0"))
data$tb_clinic_other.factor =
factor(data$tb_clinic_other,levels=c("1","0"))
data$tb_clinic_satisfaction.factor =
factor(data$tb_clinic_satisfaction,levels=c("1","2","3"))
data$tb_clinic_satisfaction_2.factor =
factor(data$tb_clinic_satisfaction_2,levels=c("1","2","3"))
data$tb_missed_clinic_date___1.factor =
factor(data$tb_missed_clinic_date___1,levels=c("0","1"))
data$tb_missed_clinic_date___2.factor =
factor(data$tb_missed_clinic_date___2,levels=c("0","1"))
data$tb_missed_clinic_date___3.factor =
factor(data$tb_missed_clinic_date___3,levels=c("0","1"))
data$tb_missedclinic_other.factor =
factor(data$tb_missedclinic_other,levels=c("1","0"))
data$missed_reason___1.factor =
factor(data$missed_reason___1,levels=c("0","1"))
data$missed_reason___2.factor =
factor(data$missed_reason___2,levels=c("0","1"))
data$missed_reason___3.factor =
factor(data$missed_reason___3,levels=c("0","1"))
data$missed_reason___4.factor =
factor(data$missed_reason___4,levels=c("0","1"))
data$missed_reason___5.factor =
factor(data$missed_reason___5,levels=c("0","1"))
data$missed_reason___6.factor =
factor(data$missed_reason___6,levels=c("0","1"))
data$missed_reason___7.factor =
factor(data$missed_reason___7,levels=c("0","1"))
data$missed_reason___8.factor =
factor(data$missed_reason___8,levels=c("0","1"))
data$missed_reason___9.factor =
factor(data$missed_reason___9,levels=c("0","1"))
data$missed_reason___10.factor =
factor(data$missed_reason___10,levels=c("0","1"))
data$missed_reason___11.factor =
factor(data$missed_reason___11,levels=c("0","1"))
data$missed_reason___12.factor =
```

```
factor(data$missed_reason___12,levels=c("0","1"))
data$missed_reason___13.factor =
factor(data$missed_reason___13,levels=c("0","1"))
data$missed_reason___14.factor =
factor(data$missed_reason___14,levels=c("0","1"))
data$tb_admitted_date.factor =
factor(data$tb_admitted_date,levels=c("1","2","3"))
data$time_to_ward.factor =
factor(data$time_to_ward,levels=c("1","2","3","4","5"))
data$tb_ward_reason___1.factor =
factor(data$tb_ward_reason___1,levels=c("0","1"))
data$tb_ward_reason___2.factor =
factor(data$tb_ward_reason___2,levels=c("0","1"))
data$tb_ward_reason___3.factor =
factor(data$tb_ward_reason___3,levels=c("0","1"))
data$tb_ward_reason___4.factor =
factor(data$tb_ward_reason___4,levels=c("0","1"))
data$tb_ward_reason___5.factor =
factor(data$tb_ward_reason___5,levels=c("0","1"))
data$tb_ward_reason___6.factor =
factor(data$tb_ward_reason___6,levels=c("0","1"))
data$tb_ward_reason___7.factor =
factor(data$tb_ward_reason___7,levels=c("0","1"))
data$tb_ward_reason___8.factor =
factor(data$tb_ward_reason___8,levels=c("0","1"))
data$tb_ward_reason___9.factor =
factor(data$tb_ward_reason___9,levels=c("0","1"))
data$tb_ward_reason___10.factor =
factor(data$tb_ward_reason___10,levels=c("0","1"))
data$tb_ward_reason___11.factor =
factor(data$tb_ward_reason___11,levels=c("0","1"))
data$tb_ward_reason___12.factor =
factor(data$tb_ward_reason___12,levels=c("0","1"))
data$tb_ward_reason___13.factor =
factor(data$tb_ward_reason___13,levels=c("0","1"))
data$tb_ward_reason___14.factor =
factor(data$tb_ward_reason___14,levels=c("0","1"))
data$tb_ward_perception.factor =
factor(data$tb_ward_perception,levels=c("1","2","3"))
data$tb_ward_perception_2.factor =
factor(data$tb_ward_perception_2,levels=c("1","2","3"))
data$tbtreatment.factor =
factor(data$tbtreatment,levels=c("0","1","2"))
data$tb_med___1.factor = factor(data$tb_med___1,levels=c("0","1"))
data$tb_med___2.factor = factor(data$tb_med___2,levels=c("0","1"))
data$tb_med___3.factor = factor(data$tb_med___3,levels=c("0","1"))
data$tb_med___4.factor = factor(data$tb_med___4,levels=c("0","1"))
data$tb_complete.factor = factor(data$tb_complete,levels=c("1","0"))
data$tb_complete_reason___1.factor =
factor(data$tb_complete_reason___1,levels=c("0","1"))
```

```
data$tb_complete_reason___2.factor =
factor(data$tb_complete_reason___2,levels=c("0","1"))
data$tb_complete_reason___3.factor =
factor(data$tb_complete_reason___3,levels=c("0","1"))
data$tb_complete_reason___4.factor =
factor(data$tb_complete_reason___4,levels=c("0","1"))
data$tb_complete_reason___5.factor =
factor(data$tb_complete_reason___5,levels=c("0","1"))
data$tuberculosis_questionnaire_complete.factor =
factor(data$tuberculosis_questionnaire_complete,levels=c("0","1","2"))
data$pmhx___1.factor = factor(data$pmhx___1,levels=c("0","1"))
data$pmhx___2.factor = factor(data$pmhx___2,levels=c("0","1"))
data$pmhx___3.factor = factor(data$pmhx___3,levels=c("0","1"))
data$pmhx___4.factor = factor(data$pmhx___4,levels=c("0","1"))
data$pmhx___5.factor = factor(data$pmhx___5,levels=c("0","1"))
data$pmhx___6.factor = factor(data$pmhx___6,levels=c("0","1"))
data$pmhx___7.factor = factor(data$pmhx___7,levels=c("0","1"))
data$pmhx___8.factor = factor(data$pmhx___8,levels=c("0","1"))
data$pmhx___9.factor = factor(data$pmhx___9,levels=c("0","1"))
data$pmhx___10.factor = factor(data$pmhx___10,levels=c("0","1"))
data$pmhx___11.factor = factor(data$pmhx___11,levels=c("0","1"))
data$pmhx___12.factor = factor(data$pmhx___12,levels=c("0","1"))
data$pmhx___13.factor = factor(data$pmhx___13,levels=c("0","1"))
data$pmhx___14.factor = factor(data$pmhx___14,levels=c("0","1"))
data$regularmeds___1.factor =
factor(data$regularmeds___1,levels=c("0","1"))
data$regularmeds___2.factor =
factor(data$regularmeds___2,levels=c("0","1"))
data$regularmeds___3.factor =
factor(data$regularmeds___3,levels=c("0","1"))
data$regularmeds___4.factor =
factor(data$regularmeds___4,levels=c("0","1"))
data$regularmeds___5.factor =
factor(data$regularmeds___5,levels=c("0","1"))
data$regularmeds___6.factor =
factor(data$regularmeds___6,levels=c("0","1"))
data$regularmeds___7.factor =
factor(data$regularmeds___7,levels=c("0","1"))
data$other_med.factor = factor(data$other_med,levels=c("1","0"))
data$exp_cig.factor = factor(data$exp_cig,levels=c("1","0"))
data$exp_cig_current.factor =
factor(data$exp_cig_current,levels=c("1","0"))
data$exp_cig_tb.factor = factor(data$exp_cig_tb,levels=c("1","0"))
data$exp_smoke.factor = factor(data$exp_smoke,levels=c("1","0"))
data$exp_pipe.factor = factor(data$exp_pipe,levels=c("1","0"))
data$exp_pipe_now.factor = factor(data$exp_pipe_now,levels=c("1","0"))
data$exp_cigar.factor = factor(data$exp_cigar,levels=c("1","0"))
data$exp_cigar_now.factor =
factor(data$exp_cigar_now,levels=c("1","0"))
data$exp_shisha.factor = factor(data$exp_shisha,levels=c("1","0"))
```

```
data$wami_sanitation.factor =
factor(data$wami_sanitation,levels=c("1","2","3","4","5","6","7","8"))
data$wami_water.factor =
factor(data$wami_water,levels=c("1","2","3","4","5","6","7","8"))
data$wami_asset_cookroom.factor =
factor(data$wami_asset_cookroom,levels=c("1","0"))
data$wami_asset_multiple___1.factor =
factor(data$wami_asset_multiple___1,levels=c("0","1"))
data$wami_asset_multiple___2.factor =
factor(data$wami_asset_multiple___2,levels=c("0","1"))
data$wami_asset_multiple___3.factor =
factor(data$wami_asset_multiple___3,levels=c("0","1"))
data$wami_asset_multiple___4.factor =
factor(data$wami_asset_multiple___4,levels=c("0","1"))
data$wami_asset_multiple___5.factor =
factor(data$wami_asset_multiple___5,levels=c("0","1"))
data$wami_asset_multiple___6.factor =
factor(data$wami_asset_multiple___6,levels=c("0","1"))
data$incometime.factor =
factor(data$incometime,levels=c("1","2","3","4"))
data$employment___1.factor =
factor(data$employment___1,levels=c("0","1"))
data$employment___2.factor =
factor(data$employment___2,levels=c("0","1"))
data$employment___3.factor =
factor(data$employment___3,levels=c("0","1"))
data$employment___4.factor =
factor(data$employment___4,levels=c("0","1"))
data$employment___5.factor =
factor(data$employment___5,levels=c("0","1"))
data$employment___6.factor =
factor(data$employment___6,levels=c("0","1"))
data$employment___7.factor =
factor(data$employment___7,levels=c("0","1"))
data$employment___8.factor =
factor(data$employment___8,levels=c("0","1"))
data$employment___9.factor =
factor(data$employment___9,levels=c("0","1"))
data$employment___10.factor =
factor(data$employment___10,levels=c("0","1"))
data$employment____99.factor =
factor(data$employment____99,levels=c("0","1"))
data$hohwork.factor = factor(data$hohwork,levels=c("1","0"))
data$marital_status.factor =
factor(data$marital_status,levels=c("1","0"))
data$cooking.factor = factor(data$cooking,levels=c("1","0"))
data$stovetype___1.factor =
factor(data$stovetype___1,levels=c("0","1"))
data$stovetype___2.factor =
factor(data$stovetype___2,levels=c("0","1"))
```

```
data$stovetype___3.factor =
factor(data$stovetype___3,levels=c("0","1"))
data$stovetype___4.factor =
factor(data$stovetype___4,levels=c("0","1"))
data$stovetype___5.factor =
factor(data$stovetype___5,levels=c("0","1"))
data$stovetype___6.factor =
factor(data$stovetype___6,levels=c("0","1"))
data$stovetype___7.factor =
factor(data$stovetype___7,levels=c("0","1"))
data$stovetype___8.factor =
factor(data$stovetype___8,levels=c("0","1"))
data$cookingarea.factor =
factor(data$cookingarea,levels=c("1","2","3"))
data$separatekitchen.factor =
factor(data$separatekitchen,levels=c("1","0"))
data$fuelsource.factor =
factor(data$fuelsource,levels=c("1","2","3","4","5","6","7","8","9","1
0","11","12"))
data$biomass.factor = factor(data$biomass,levels=c("1","0"))
data$biofuels___1.factor = factor(data$biofuels___1,levels=c("0","1"))
data$biofuels___2.factor = factor(data$biofuels___2,levels=c("0","1"))
data$biofuels___3.factor = factor(data$biofuels___3,levels=c("0","1"))
data$biofuels___4.factor = factor(data$biofuels___4,levels=c("0","1"))
data$biofuels___5.factor = factor(data$biofuels___5,levels=c("0","1"))
data$pastbiomass.factor = factor(data$pastbiomass,levels=c("1","0"))
data$idi.factor = factor(data$idi,levels=c("1","0"))
data$tb_complete_2.factor =
factor(data$tb_complete_2,levels=c("1","0"))
data$tb_complete_reason_2___1.factor =
factor(data$tb_complete_reason_2___1,levels=c("0","1"))
data$tb_complete_reason_2___2.factor =
factor(data$tb_complete_reason_2___2,levels=c("0","1"))
data$tb_complete_reason_2___3.factor =
factor(data$tb_complete_reason_2___3,levels=c("0","1"))
data$tb_complete_reason_2___4.factor =
factor(data$tb_complete_reason_2___4,levels=c("0","1"))
data$tb_complete_reason_2___5.factor =
factor(data$tb_complete_reason_2___5,levels=c("0","1"))
data$phone_questionnaire_general_complete.factor =
factor(data$phone_questionnaire_general_complete,levels=c("0","1","2")
)
data$everwheeze.factor = factor(data$everwheeze,levels=c("1","0"))
data$recentwheeze.factor = factor(data$recentwheeze,levels=c("1","0"))
data$seekcare.factor =
factor(data$seekcare,levels=c("1","2","3","4","5"))
data$cough.factor = factor(data$cough,levels=c("1","0"))
data$cbronch.factor = factor(data$cbronch,levels=c("1","0"))
data$hospitalized.factor = factor(data$hospitalized,levels=c("1","0"))
data$impeded.factor = factor(data$impeded,levels=c("1","0"))
```

```
data$cap5.factor = factor(data$cap5,levels=c("1","2","3"))
data$eq1.factor = factor(data$eq1,levels=c("1","2","3"))
data$eq2.factor = factor(data$eq2,levels=c("1","2","3"))
data$eq3.factor = factor(data$eq3,levels=c("1","2","3"))
data$eq4.factor = factor(data$eq4,levels=c("1","2","3"))
data$eq5.factor = factor(data$eq5,levels=c("1","2","3"))
data$tb_complete_3.factor =
factor(data$tb_complete_3,levels=c("1","0"))
data$tb_complete_reason_3___1.factor =
factor(data$tb_complete_reason_3___1,levels=c("0","1"))
data$tb_complete_reason_3___2.factor =
factor(data$tb_complete_reason_3___2,levels=c("0","1"))
data$tb_complete_reason_3___3.factor =
factor(data$tb_complete_reason_3___3,levels=c("0","1"))
data$tb_complete_reason_3___4.factor =
factor(data$tb_complete_reason_3___4,levels=c("0","1"))
data$tb_complete_reason_3___5.factor =
factor(data$tb_complete_reason_3___5,levels=c("0","1"))
data$respiratory_and_eq5d3l_complete.factor =
factor(data$respiratory_and_eq5d3l_complete,levels=c("0","1","2"))
data$phq1.factor = factor(data$phq1,levels=c("0","1","2","3"))
data$phq2.factor = factor(data$phq2,levels=c("0","1","2","3"))
data$phq3.factor = factor(data$phq3,levels=c("0","1","2","3"))
data$phq4.factor = factor(data$phq4,levels=c("0","1","2","3"))
data$phq5.factor = factor(data$phq5,levels=c("0","1","2","3"))
data$phq6.factor = factor(data$phq6,levels=c("0","1","2","3"))
data$phq7.factor = factor(data$phq7,levels=c("0","1","2","3"))
data$phq8.factor = factor(data$phq8,levels=c("0","1","2","3"))
data$phq9.factor = factor(data$phq9,levels=c("0","1","2","3"))
data$phq10.factor = factor(data$phq10,levels=c("0","1","2","3"))
data$tb_complete_4.factor =
factor(data$tb_complete_4,levels=c("1","0"))
data$tb_complete_reason_4___1.factor =
factor(data$tb_complete_reason_4___1,levels=c("0","1"))
data$tb_complete_reason_4___2.factor =
factor(data$tb_complete_reason_4___2,levels=c("0","1"))
data$tb_complete_reason_4___3.factor =
factor(data$tb_complete_reason_4___3,levels=c("0","1"))
data$tb_complete_reason_4___4.factor =
factor(data$tb_complete_reason_4___4,levels=c("0","1"))
data$tb_complete_reason_4___5.factor =
factor(data$tb_complete_reason_4___5,levels=c("0","1"))
data$phq9_complete.factor =
factor(data$phq9_complete,levels=c("0","1","2"))
data$sgrq_current_health.factor =
factor(data$sgrq_current_health,levels=c("1","2","3","4","5"))
data$sgrq_1.factor = factor(data$sgrq_1,levels=c("1","2","3","4","5"))
data$sgrq_2.factor = factor(data$sgrq_2,levels=c("1","2","3","4","5"))
data$sgrq_3.factor = factor(data$sgrq_3,levels=c("1","2","3","4","5"))
data$sgrq_4.factor = factor(data$sgrq_4,levels=c("1","2","3","4","5"))
```

```
data$sgrq_5.factor = factor(data$sgrq_5,levels=c("1","2","3","4","5"))
data$sgrq_6.factor = factor(data$sgrq_6,levels=c("1","2","3","4"))
data$sgrq_7.factor = factor(data$sgrq_7,levels=c("1","2","3","4","5"))
data$sgrq_8.factor = factor(data$sgrq_8,levels=c("1","0"))
data$sgrq_sect_1_1.factor =
factor(data$sgrq_sect_1_1,levels=c("1","2","3","4"))
data$sgrq_sect_1_2.factor =
factor(data$sgrq_sect_1_2,levels=c("1","2","3"))
data$sgrq_sect_2_1.factor =
factor(data$sgrq_sect_2_1,levels=c("1","0"))
data$sgrq_sect_2_2.factor =
factor(data$sgrq_sect_2_2,levels=c("1","0"))
data$sgrq_sect_2_3.factor =
factor(data$sgrq_sect_2_3,levels=c("1","0"))
data$sgrq_sect_2_4.factor =
factor(data$sgrq_sect_2_4,levels=c("1","0"))
data$sgrq_sect_2_5.factor =
factor(data$sgrq_sect_2_5,levels=c("1","0"))
data$sgrq_sect_2_6.factor =
factor(data$sgrq_sect_2_6,levels=c("1","0"))
data$sgrq_sect_2_7.factor =
factor(data$sgrq_sect_2_7,levels=c("1","0"))
data$sgrq_sect_3_1.factor =
factor(data$sgrq_sect_3_1,levels=c("1","0"))
data$sgrq_sect_3_2.factor =
factor(data$sgrq_sect_3_2,levels=c("1","0"))
data$sgrq_sect_3_3.factor =
factor(data$sgrq_sect_3_3,levels=c("1","0"))
data$sgrq_sect_3_4.factor =
factor(data$sgrq_sect_3_4,levels=c("1","0"))
data$sgrq_sect_3_5.factor =
factor(data$sgrq_sect_3_5,levels=c("1","0"))
data$sgrq_sect_3_6.factor =
factor(data$sgrq_sect_3_6,levels=c("1","0"))
data$sgrq_sect_4_1.factor =
factor(data$sgrq_sect_4_1,levels=c("1","0"))
data$sgrq_sect_4_2.factor =
factor(data$sgrq_sect_4_2,levels=c("1","0"))
data$sgrq_sect_4_3.factor =
factor(data$sgrq_sect_4_3,levels=c("1","0"))
data$sgrq_sect_4_4.factor =
factor(data$sgrq_sect_4_4,levels=c("1","0"))
data$sgrq_sect_4_5.factor =
factor(data$sgrq_sect_4_5,levels=c("1","0"))
data$sgrq_sect_4_6.factor =
factor(data$sgrq_sect_4_6,levels=c("1","0"))
data$sgrq_sect_4_7.factor =
factor(data$sgrq_sect_4_7,levels=c("1","0"))
data$sgrq_sect_4_8.factor =
factor(data$sgrq_sect_4_8,levels=c("1","0"))
```

```
data$sgrq_sec_5_0.factor = factor(data$sgrq_sec_5_0,levels=c("1","0"))
data$sgrq_sect_5_1.factor =
factor(data$sgrq_sect_5_1,levels=c("1","0"))
data$sgrq_sect_5_2.factor =
factor(data$sgrq_sect_5_2,levels=c("1","0"))
data$sgrq_sect_5_3.factor =
factor(data$sgrq_sect_5_3,levels=c("1","0"))
data$sgrq_sect_5_4.factor =
factor(data$sgrq_sect_5_4,levels=c("1","0"))
data$sgrq_sect_6_1.factor =
factor(data$sgrq_sect_6_1,levels=c("1","0"))
data$sgrq_sect_6_2.factor =
factor(data$sgrq_sect_6_2,levels=c("1","0"))
data$sgrq_sect_6_3.factor =
factor(data$sgrq_sect_6_3,levels=c("1","0"))
data$sgrq_sect_6_4.factor =
factor(data$sgrq_sect_6_4,levels=c("1","0"))
data$sgrq_sect_6_5.factor =
factor(data$sgrq_sect_6_5,levels=c("1","0"))
data$sgrq_sect_6_6.factor =
factor(data$sgrq_sect_6_6,levels=c("1","0"))
data$sgrq_sect_6_7.factor =
factor(data$sgrq_sect_6_7,levels=c("1","0"))
data$sgrq_sect_6_8.factor =
factor(data$sgrq_sect_6_8,levels=c("1","0"))
data$sgrq_sect_6_9.factor =
factor(data$sgrq_sect_6_9,levels=c("1","0"))
data$sgrq_sect_7_1.factor =
factor(data$sgrq_sect_7_1,levels=c("1","0"))
data$sgrq_sect_7_2.factor =
factor(data$sgrq_sect_7_2,levels=c("1","0"))
data$sgrq_sect_7_3.factor =
factor(data$sgrq_sect_7_3,levels=c("1","0"))
data$sgrq_sect_7_4.factor =
factor(data$sgrq_sect_7_4,levels=c("1","0"))
data$sgrq_sect_7_5.factor =
factor(data$sgrq_sect_7_5,levels=c("1","0"))
data$sgrq_affects.factor =
factor(data$sgrq_affects,levels=c("1","2","3","4"))
data$tb_complete_5.factor =
factor(data$tb_complete_5,levels=c("1","0"))
data$tb_complete_reason_5___1.factor =
factor(data$tb_complete_reason_5___1,levels=c("0","1"))
data$tb_complete_reason_5___2.factor =
factor(data$tb_complete_reason_5___2,levels=c("0","1"))
data$tb_complete_reason_5___3.factor =
factor(data$tb_complete_reason_5___3,levels=c("0","1"))
data$tb_complete_reason_5___4.factor =
factor(data$tb_complete_reason_5___4,levels=c("0","1"))
data$tb_complete_reason_5___5.factor =
```

```
factor(data$tb_complete_reason_5___5,levels=c("0","1"))
data$appendix1_st_georges_respiratory_questionnaire_sgr_complete.facto
r =
factor(data$appendix1_st_georges_respiratory_questionnaire_sgr_complet
e,levels=c("0","1","2"))

levels(data$redcap_event_name.factor)=c("Screening Data","Study Data")
levels(data$study_site.factor)=c("Jinja Referral Hospital","Mubende
Regional Referral hospital","Kiboga General Hospital","Kiruddu
National Referral Hospital","Kisenyi Health Center IV","Mulago
National Referral Hospital","Other or multiple")
levels(data$tb_setting_all_d.factor)=c("Diagnosed with
tuberculosis","Visited tuberculosis clinic","Missed a tuberculosis
clinic appointment","Admitted to tuberculosis inpatient ward")
levels(data$tb_dx_time_period_d.factor)=c("September, 2019 - March,
15th, 2020","March 15th - June, 2020","After June 2020","June 1st -
July 31, 2021")
levels(data$sex.factor)=c("Female","Male")
levels(data$mobilephone.factor)=c("Yes","No")
levels(data$contact.factor)=c("Yes","No")
levels(data$relationship.factor)=c("Parent","Child","Sibling","Partner
","Other family","Friend","Other (Not Specified)")
levels(data$demographics_complete.factor)=c("Incomplete","Unverified",
"Complete")
levels(data$alive.factor)=c("Yes","No")
levels(data$consent_read.factor)=c("Yes","No")
levels(data$consent_obtained.factor)=c("Yes","No")
levels(data$consent_complete.factor)=c("Incomplete","Unverified","Comp
lete")
levels(data$tb_setting_all___1.factor)=c("Unchecked","Checked")
levels(data$tb_setting_all___2.factor)=c("Unchecked","Checked")
levels(data$tb_setting_all___3.factor)=c("Unchecked","Checked")
levels(data$tb_setting_all___4.factor)=c("Unchecked","Checked")
levels(data$tb_multiple.factor)=c("Yes","No")
levels(data$tb_dx_time_period.factor)=c("September, 2019 - March,
15th, 2020","March 15th - June, 2020","After June 2020")
levels(data$placediagtb.factor)=c("Community care facility","Primary
care or clinic","Public care facility","Private Health
Facility","Hospital","Other")
levels(data$tb_dx_site.factor)=c("Jinja Regional Referral
Hospital","Mubende Regional Referral Hospital","Kiboga General
Hospital","Kiruddu National Referral Hospital","Kisenyi Health Center
IV - Kampala","Mulago National Referral Hospital","Other")
levels(data$time_to_dx.factor)=c("Less than a week","1-2 weeks","2
weeks to a month","1-2 months","over 2 months")
levels(data$tb_dx_barrier___1.factor)=c("Unchecked","Checked")
levels(data$tb_dx_barrier___2.factor)=c("Unchecked","Checked")
levels(data$tb_dx_barrier___3.factor)=c("Unchecked","Checked")
levels(data$tb_dx_barrier___4.factor)=c("Unchecked","Checked")
levels(data$tb_dx_barrier___5.factor)=c("Unchecked","Checked")
```

```
levels(data$tb_dx_barrier___6.factor)=c("Unchecked","Checked")
levels(data$tb_dx_barrier___7.factor)=c("Unchecked","Checked")
levels(data$tb_dx_barrier___8.factor)=c("Unchecked","Checked")
levels(data$tb_dx_barrier___9.factor)=c("Unchecked","Checked")
levels(data$tb_dx_barrier___10.factor)=c("Unchecked","Checked")
levels(data$tb_dx_barrier___11.factor)=c("Unchecked","Checked")
levels(data$tb_dx_barrier___12.factor)=c("Unchecked","Checked")
levels(data$tb_dx_barrier___13.factor)=c("Unchecked","Checked")
levels(data$tb_dx_barrier___14.factor)=c("Unchecked","Checked")
levels(data$tb_clinic_timeperiod___1.factor)=c("Unchecked","Checked")
levels(data$tb_clinic_timeperiod___2.factor)=c("Unchecked","Checked")
levels(data$tb_clinic_timeperiod___3.factor)=c("Unchecked","Checked")
levels(data$tb_clinic_timeperiod___4.factor)=c("Unchecked","Checked")
levels(data$tp_clnic_complete.factor)=c("Prior to March 15th,
2020","March 15th-June, 2020","After June, 2020","Patient is still
attending TB clinic")
levels(data$missedclinic_lockdown.factor)=c("Yes","No")
levels(data$tb_clinic_other.factor)=c("Yes","No")
levels(data$tb_clinic_satisfaction.factor)=c("Yes","No","Not sure")
levels(data$tb_clinic_satisfaction_2.factor)=c("Yes","No","Not sure")
levels(data$tb_missed_clinic_date___1.factor)=c("Unchecked","Checked")
levels(data$tb_missed_clinic_date___2.factor)=c("Unchecked","Checked")
levels(data$tb_missed_clinic_date___3.factor)=c("Unchecked","Checked")
levels(data$tb_missedclinic_other.factor)=c("Yes","No")
levels(data$missed_reason___1.factor)=c("Unchecked","Checked")
levels(data$missed_reason___2.factor)=c("Unchecked","Checked")
levels(data$missed_reason___3.factor)=c("Unchecked","Checked")
levels(data$missed_reason___4.factor)=c("Unchecked","Checked")
levels(data$missed_reason___5.factor)=c("Unchecked","Checked")
levels(data$missed_reason___6.factor)=c("Unchecked","Checked")
levels(data$missed_reason___7.factor)=c("Unchecked","Checked")
levels(data$missed_reason___8.factor)=c("Unchecked","Checked")
levels(data$missed_reason___9.factor)=c("Unchecked","Checked")
levels(data$missed_reason___10.factor)=c("Unchecked","Checked")
levels(data$missed_reason___11.factor)=c("Unchecked","Checked")
levels(data$missed_reason___12.factor)=c("Unchecked","Checked")
levels(data$missed_reason___13.factor)=c("Unchecked","Checked")
levels(data$missed_reason___14.factor)=c("Unchecked","Checked")
levels(data$tb_admitted_date.factor)=c("Prior to March, 15th,
2020","March 15th-June, 2020","After June, 2020")
levels(data$time_to_ward.factor)=c("Less than a week","1-2 weeks","2
weeks to a month","1-2 months","over 2 months")
levels(data$tb_ward_reason___1.factor)=c("Unchecked","Checked")
levels(data$tb_ward_reason___2.factor)=c("Unchecked","Checked")
levels(data$tb_ward_reason___3.factor)=c("Unchecked","Checked")
levels(data$tb_ward_reason___4.factor)=c("Unchecked","Checked")
levels(data$tb_ward_reason___5.factor)=c("Unchecked","Checked")
levels(data$tb_ward_reason___6.factor)=c("Unchecked","Checked")
levels(data$tb_ward_reason___7.factor)=c("Unchecked","Checked")
levels(data$tb_ward_reason___8.factor)=c("Unchecked","Checked")
```

```
levels(data$tb_ward_reason___9.factor)=c("Unchecked","Checked")
levels(data$tb_ward_reason___10.factor)=c("Unchecked","Checked")
levels(data$tb_ward_reason___11.factor)=c("Unchecked","Checked")
levels(data$tb_ward_reason___12.factor)=c("Unchecked","Checked")
levels(data$tb_ward_reason___13.factor)=c("Unchecked","Checked")
levels(data$tb_ward_reason___14.factor)=c("Unchecked","Checked")
levels(data$tb_ward_perception.factor)=c("Yes","No","Unsure")
levels(data$tb_ward_perception_2.factor)=c("Yes","No","Unsure")
levels(data$tbtreatment.factor)=c("No, did not complete
treatment","No, currently receiving treatment","Yes, completed
treatment")
levels(data$tb_med___1.factor)=c("Unchecked","Checked")
levels(data$tb_med___2.factor)=c("Unchecked","Checked")
levels(data$tb_med___3.factor)=c("Unchecked","Checked")
levels(data$tb_med___4.factor)=c("Unchecked","Checked")
levels(data$tb_complete.factor)=c("Yes","No")
levels(data$tb_complete_reason___1.factor)=c("Unchecked","Checked")
levels(data$tb_complete_reason___2.factor)=c("Unchecked","Checked")
levels(data$tb_complete_reason___3.factor)=c("Unchecked","Checked")
levels(data$tb_complete_reason___4.factor)=c("Unchecked","Checked")
levels(data$tb_complete_reason___5.factor)=c("Unchecked","Checked")
levels(data$tuberculosis_questionnaire_complete.factor)=c("Incomplete"
,"Unverified","Complete")
levels(data$pmhx___1.factor)=c("Unchecked","Checked")
levels(data$pmhx___2.factor)=c("Unchecked","Checked")
levels(data$pmhx___3.factor)=c("Unchecked","Checked")
levels(data$pmhx___4.factor)=c("Unchecked","Checked")
levels(data$pmhx___5.factor)=c("Unchecked","Checked")
levels(data$pmhx___6.factor)=c("Unchecked","Checked")
levels(data$pmhx___7.factor)=c("Unchecked","Checked")
levels(data$pmhx___8.factor)=c("Unchecked","Checked")
levels(data$pmhx___9.factor)=c("Unchecked","Checked")
levels(data$pmhx___10.factor)=c("Unchecked","Checked")
levels(data$pmhx___11.factor)=c("Unchecked","Checked")
levels(data$pmhx___12.factor)=c("Unchecked","Checked")
levels(data$pmhx___13.factor)=c("Unchecked","Checked")
levels(data$pmhx___14.factor)=c("Unchecked","Checked")
levels(data$regularmeds___1.factor)=c("Unchecked","Checked")
levels(data$regularmeds___2.factor)=c("Unchecked","Checked")
levels(data$regularmeds___3.factor)=c("Unchecked","Checked")
levels(data$regularmeds___4.factor)=c("Unchecked","Checked")
levels(data$regularmeds___5.factor)=c("Unchecked","Checked")
levels(data$regularmeds___6.factor)=c("Unchecked","Checked")
levels(data$regularmeds___7.factor)=c("Unchecked","Checked")
levels(data$other_med.factor)=c("Yes","No")
levels(data$exp_cig.factor)=c("Yes","No")
levels(data$exp_cig_current.factor)=c("Yes","No")
levels(data$exp_cig_tb.factor)=c("Yes","No")
levels(data$exp_smoke.factor)=c("Yes","No")
levels(data$exp_pipe.factor)=c("Yes","No")
```

```
levels(data$exp_pipe_now.factor)=c("Yes","No")
levels(data$exp_cigar.factor)=c("Yes","No")
levels(data$exp_cigar_now.factor)=c("Yes","No")
levels(data$exp_shisha.factor)=c("Yes","No")
levels(data$wami_sanitation.factor)=c("No facility/bush/field or
bucket toilet","Pit latrine without flush (with slab)","Flush to piped
sewer system","Flush to septic tank","Flush to pit latrine","Flush to
somewhere else","Pit latrine without slab","Other")
levels(data$wami_water.factor)=c("Piped into dwelling","Piped to yard/
plot","Public tap/stand pipe","Tube well or borehole","Protected
well","Unprotected well","Surface water (river, dam, lake, pond,
stream, canal, irrigation canal)","Other")
levels(data$wami_asset_cookroom.factor)=c("Yes","No")
levels(data$wami_asset_multiple___1.factor)=c("Unchecked","Checked")
levels(data$wami_asset_multiple___2.factor)=c("Unchecked","Checked")
levels(data$wami_asset_multiple___3.factor)=c("Unchecked","Checked")
levels(data$wami_asset_multiple___4.factor)=c("Unchecked","Checked")
levels(data$wami_asset_multiple___5.factor)=c("Unchecked","Checked")
levels(data$wami_asset_multiple___6.factor)=c("Unchecked","Checked")
levels(data$incometime.factor)=c("Day","Week","Month","Year")
levels(data$employment___1.factor)=c("Unchecked","Checked")
levels(data$employment___2.factor)=c("Unchecked","Checked")
levels(data$employment___3.factor)=c("Unchecked","Checked")
levels(data$employment___4.factor)=c("Unchecked","Checked")
levels(data$employment___5.factor)=c("Unchecked","Checked")
levels(data$employment___6.factor)=c("Unchecked","Checked")
levels(data$employment___7.factor)=c("Unchecked","Checked")
levels(data$employment___8.factor)=c("Unchecked","Checked")
levels(data$employment___9.factor)=c("Unchecked","Checked")
levels(data$employment___10.factor)=c("Unchecked","Checked")
levels(data$employment____99.factor)=c("Unchecked","Checked")
levels(data$hohwork.factor)=c("Yes","No")
levels(data$marital_status.factor)=c("Yes","No")
levels(data$cooking.factor)=c("Yes","No")
levels(data$stovetype___1.factor)=c("Unchecked","Checked")
levels(data$stovetype___2.factor)=c("Unchecked","Checked")
levels(data$stovetype___3.factor)=c("Unchecked","Checked")
levels(data$stovetype___4.factor)=c("Unchecked","Checked")
levels(data$stovetype___5.factor)=c("Unchecked","Checked")
levels(data$stovetype___6.factor)=c("Unchecked","Checked")
levels(data$stovetype___7.factor)=c("Unchecked","Checked")
levels(data$stovetype___8.factor)=c("Unchecked","Checked")
levels(data$cookingarea.factor)=c("Inside the house","Outside the
house","Both inside and outside the house")
levels(data$separatekitchen.factor)=c("Yes","No")
levels(data$fuelsource.factor)=c("Electricity","Liquified petroleum
gas (LPG)","Natural gas","Biogas","Kerosene","Coal/
Ignite","Charcoal","Wood","Straw/shrubs/grass","Agricultural
crop","Animal dung","Other")
levels(data$biomass.factor)=c("Yes","No")
```

```
levels(data$biofuels___1.factor)=c("Unchecked","Checked")
levels(data$biofuels___2.factor)=c("Unchecked","Checked")
levels(data$biofuels___3.factor)=c("Unchecked","Checked")
levels(data$biofuels___4.factor)=c("Unchecked","Checked")
levels(data$biofuels___5.factor)=c("Unchecked","Checked")
levels(data$pastbiomass.factor)=c("Yes","No")
levels(data$idi.factor)=c("Yes","No")
levels(data$tb_complete_2.factor)=c("Yes","No")
levels(data$tb_complete_reason_2___1.factor)=c("Unchecked","Checked")
levels(data$tb_complete_reason_2___2.factor)=c("Unchecked","Checked")
levels(data$tb_complete_reason_2___3.factor)=c("Unchecked","Checked")
levels(data$tb_complete_reason_2___4.factor)=c("Unchecked","Checked")
levels(data$tb_complete_reason_2___5.factor)=c("Unchecked","Checked")
levels(data$phone_questionnaire_general_complete.factor)=c("Incomplete
","Unverified","Complete")
levels(data$everwheeze.factor)=c("Yes","No")
levels(data$recentwheeze.factor)=c("Yes","No")
levels(data$seekcare.factor)=c("I do not seek care","Local
hospital","Local health clinic","Informal local healer","Other")
levels(data$cough.factor)=c("Yes","No")
levels(data$cbronch.factor)=c("Yes","No")
levels(data$hospitalized.factor)=c("Yes","No")
levels(data$impeded.factor)=c("Yes","No")
levels(data$cap5.factor)=c("Zero","One","Two or more")
levels(data$eq1.factor)=c("I have no problems in walking about (Sirina
buzibu mu kutambula)","I have some problems in walking about (Nina mu
obuzibuzibu mukutambula)","I am confined to bed (Siva mu buliri)")
levels(data$eq2.factor)=c("I have no problems with self-care (Sirina
buzibu mu kwefaako)","I have some problems washing or dressing myself
(Nina mu obuzibuzibu mukwenaaza oba okweyambaza)","I am unable to wash
or dress myself (Sisobolera ddala kwenaaza oba kweyambaza)")
levels(data$eq3.factor)=c("I have no problems with performing my usual
activities (Sirina buzibu mukwekolera bintu byange ebya bulijjo)","I
have some problems with performing my usual activities (Nina mu
obuzibuzibu mukwekolera ebintu byange ebya bulijjo)","I am unable to
perform my usual activities (Sisobola kwekolera bintu byange ebya
bulijjo)")
levels(data$eq4.factor)=c("I have no pain or discomfort (Sirina bulumi
oba butewulira bulungi)","I have moderate pain or discomfort (Nina
obulumi obuwerako oba obutewulira bulungi obuwerako)","I have extreme
pain or discomfort (Nina obulumi bungi nnyo nnnyo oba obutewulira
bulungi bungi nnyo nnyo)")
levels(data$eq5.factor)=c("I am not anxious or depressed (Siri
mwelarikirivu oba kuba na nyiike)","I am moderately anxious or
depressed (Nina obwelarikirivu bwakigero oba enyiike yakigero)","I am
extremely anxious or depressed (Ndimwelarikirivu nnyo nnyo oba nina
enyiike nyingi nnyo nnyo)")
levels(data$tb_complete_3.factor)=c("Yes","No")
levels(data$tb_complete_reason_3___1.factor)=c("Unchecked","Checked")
levels(data$tb_complete_reason_3___2.factor)=c("Unchecked","Checked")
```

```
levels(data$tb_complete_reason_3___3.factor)=c("Unchecked","Checked")
levels(data$tb_complete_reason_3___4.factor)=c("Unchecked","Checked")
levels(data$tb_complete_reason_3___5.factor)=c("Unchecked","Checked")
levels(data$respiratory_and_eq5d3l_complete.factor)=c("Incomplete","Un
verified","Complete")
levels(data$phq1.factor)=c("Not at all","Several days","More than half
the days","Nearly every day")
levels(data$phq2.factor)=c("Not at all","Several days","More than half
the days","Nearly every day")
levels(data$phq3.factor)=c("Not at all","Several days","More than half
the days","Nearly every day")
levels(data$phq4.factor)=c("Not at all","Several days","More than half
the days","Nearly every day")
levels(data$phq5.factor)=c("Not at all","Several days","More than half
the days","Nearly every day")
levels(data$phq6.factor)=c("Not at all","Several days","More than half
the days","Nearly every day")
levels(data$phq7.factor)=c("Not at all","Several days","More than half
the days","Nearly every day")
levels(data$phq8.factor)=c("Not at all","Several days","More than half
the days","Nearly every day")
levels(data$phq9.factor)=c("Not at all","Several days","More than half
the days","Nearly every day")
levels(data$phq10.factor)=c("Not difficult at all","Somewhat
difficult","Very difficult","Extremely difficult")
levels(data$tb_complete_4.factor)=c("Yes","No")
levels(data$tb_complete_reason_4___1.factor)=c("Unchecked","Checked")
levels(data$tb_complete_reason_4___2.factor)=c("Unchecked","Checked")
levels(data$tb_complete_reason_4___3.factor)=c("Unchecked","Checked")
levels(data$tb_complete_reason_4___4.factor)=c("Unchecked","Checked")
levels(data$tb_complete_reason_4___5.factor)=c("Unchecked","Checked")
levels(data$phq9_complete.factor)=c("Incomplete","Unverified","Complet
e")
levels(data$sgrq_current_health.factor)=c("Very
good","Good","Fair","Poor","Very poor")
levels(data$sgrq_1.factor)=c("most days a week / Ennaku ezisinga mu
wiiki","several days a week / Ennaku eziwerako mu wiiki","a few days a
month / Ennaku entono mu mwezi","only with chest infections / Okujjako
nga nfunye ekifuba","not at all / Nedda")
levels(data$sgrq_2.factor)=c("most days a week / Ennaku ezisinga mu
wiiki","several days a week / Ennaku eziwerako mu wiiki","a few days a
month / Ennaku entono mu mwezi","only with chest infections / Okujjako
nga nfunye ekifuba","not at all / Nedda")
levels(data$sgrq_3.factor)=c("most days a week / Ennaku ezisinga mu
wiiki","several days a week / Ennaku eziwerako mu wiiki","a few days a
month / Ennaku entono mu mwezi","only with chest infections / Okujjako
nga nfunye ekifuba","not at all / Nedda")
levels(data$sgrq_4.factor)=c("most days a week / Ennaku ezisinga mu
wiiki","several days a week / Ennaku eziwerako mu wiiki","a few days a
month / Ennaku entono mu mwezi","only with chest infections / Okujjako
```

```
nga nfunye ekifuba","not at all / Nedda")
levels(data$sgrq_5.factor)=c("more than 3 attacks / Emirundi 3
nokusingawo","3 attacks / Emirundi 3","2 attacks / Emirundi 2","1
attack / Emirundi 1","no attacks / Safuna")
levels(data$sgrq_6.factor)=c("a week or more / Wiiki 1 nkusingawo","3
or more days / Ennaku 3 n_kusingawo","1 or 2 days / Olunaku 1 oba
2","less than a day / Kitundu kya lunaku")
levels(data$sgrq_7.factor)=c("No good days / Tewali","1 or 2 good
days / 1 oba 2","3 or 4 good days / 3 oba 4","nearly every day is
good / Kumpi buli lunaku","every day is good / Buli lunaku")
levels(data$sgrq_8.factor)=c("Yes","No")
levels(data$sgrq_sect_1_1.factor)=c("The most important problem I
have / Kyekizibu kyensinga okufuna","Causes me quite a lot of
problems / Kimpa obuzibu obuwelako","Causes me a few problems / Kimpa
obuzibu butono","Causes no problem / Tekimpa buzibu")
levels(data$sgrq_sect_1_2.factor)=c("My chest trouble made me stop
work altogether / Ekifuba kyandetera okulekera awo okukola","My chest
trouble interferes with my work or made me change my work / Ekifuba
kintawanya nga nkola/kyandetera okukyusa omulimu","My chest trouble
does not affect my work / Tekikosa mirimu gyange")
levels(data$sgrq_sect_2_1.factor)=c("True / Kituufu","False /
Sikituufu")
levels(data$sgrq_sect_2_2.factor)=c("True / Kituufu","False /
Sikituufu")
levels(data$sgrq_sect_2_3.factor)=c("True / Kituufu","False /
Sikituufu")
levels(data$sgrq_sect_2_4.factor)=c("True / Kituufu","False /
Sikituufu")
levels(data$sgrq_sect_2_5.factor)=c("True / Kituufu","False /
Sikituufu")
levels(data$sgrq_sect_2_6.factor)=c("True / Kituufu","False /
Sikituufu")
levels(data$sgrq_sect_2_7.factor)=c("True / Kituufu","False /
Sikituufu")
levels(data$sgrq_sect_3_1.factor)=c("True / Kituufu","False /
Sikituufu")
levels(data$sgrq_sect_3_2.factor)=c("True / Kituufu","False /
Sikituufu")
levels(data$sgrq_sect_3_3.factor)=c("True / Kituufu","False /
Sikituufu")
levels(data$sgrq_sect_3_4.factor)=c("True / Kituufu","False /
Sikituufu")
levels(data$sgrq_sect_3_5.factor)=c("True / Kituufu","False /
Sikituufu")
levels(data$sgrq_sect_3_6.factor)=c("True / Kituufu","False /
Sikituufu")
levels(data$sgrq_sect_4_1.factor)=c("True / Kituufu","False /
Sikituufu")
levels(data$sgrq_sect_4_2.factor)=c("True / Kituufu","False /
Sikituufu")
```

```
levels(data$sgrq_sect_4_3.factor)=c("True / Kituufu","False /
Sikituufu")
levels(data$sgrq_sect_4_4.factor)=c("True / Kituufu","False /
Sikituufu")
levels(data$sgrq_sect_4_5.factor)=c("True / Kituufu","False /
Sikituufu")
levels(data$sgrq_sect_4_6.factor)=c("True / Kituufu","False /
Sikituufu")
levels(data$sgrq_sect_4_7.factor)=c("True / Kituufu","False /
Sikituufu")
levels(data$sgrq_sect_4_8.factor)=c("True / Kituufu","False /
Sikituufu")
levels(data$sgrq_sec_5_0.factor)=c("True / Kituufu","False /
Sikituufu")
levels(data$sgrq_sect_5_1.factor)=c("True / Kituufu","False /
Sikituufu")
levels(data$sgrq_sect_5_2.factor)=c("True / Kituufu","False /
Sikituufu")
levels(data$sgrq_sect_5_3.factor)=c("True / Kituufu","False /
Sikituufu")
levels(data$sgrq_sect_5_4.factor)=c("True / Kituufu","False /
Sikituufu")
levels(data$sgrq_sect_6_1.factor)=c("True / Kituufu","False /
Sikituufu")
levels(data$sgrq_sect_6_2.factor)=c("True / Kituufu","False /
Sikituufu")
levels(data$sgrq_sect_6_3.factor)=c("True / Kituufu","False /
Sikituufu")
levels(data$sgrq_sect_6_4.factor)=c("True / Kituufu","False /
Sikituufu")
levels(data$sgrq_sect_6_5.factor)=c("True / Kituufu","False /
Sikituufu")
levels(data$sgrq_sect_6_6.factor)=c("True / Kituufu","False /
Sikituufu")
levels(data$sgrq_sect_6_7.factor)=c("True / Kituufu","False /
Sikituufu")
levels(data$sgrq_sect_6_8.factor)=c("True / Kituufu","False /
Sikituufu")
levels(data$sgrq_sect_6_9.factor)=c("True / Kituufu","False /
Sikituufu")
levels(data$sgrq_sect_7_1.factor)=c("True / Kituufu","False /
Sikituufu")
levels(data$sgrq_sect_7_2.factor)=c("True / Kituufu","False /
Sikituufu")
levels(data$sgrq_sect_7_3.factor)=c("True / Kituufu","False /
Sikituufu")
levels(data$sgrq_sect_7_4.factor)=c("True / Kituufu","False /
Sikituufu")
levels(data$sgrq_sect_7_5.factor)=c("True / Kituufu","False /
Sikituufu")
```

```
levels(data$sgrq_affects.factor)=c("It does not stop me doing anything
I would like to do / Tekilina kyekinemesa kukola","It stops me doing
one or two things I would like to do / Kinemesa okukola ekintu ekimu
oba biibiri byenjagala okukola","It stops me doing most of the things
I would like to do / Kinemesa okukola ebintu ebisinga byenjagala
okukola","It stops me doing everything I would like to do / Kinemesa
okukola buli kimu kyenjagala okukola")
levels(data$tb_complete_5.factor)=c("Yes","No")
levels(data$tb_complete_reason_5___1.factor)=c("Unchecked","Checked")
levels(data$tb_complete_reason_5___2.factor)=c("Unchecked","Checked")
levels(data$tb_complete_reason_5___3.factor)=c("Unchecked","Checked")
levels(data$tb_complete_reason_5___4.factor)=c("Unchecked","Checked")
levels(data$tb_complete_reason_5___5.factor)=c("Unchecked","Checked")
levels(data$appendix1_st_georges_respiratory_questionnaire_sgr_complet
e.factor)=c("Incomplete","Unverified","Complete")
```
