# Supplementary material for: The effect of biomass smoke exposure on quality-of-life among Ugandan patients treated for tuberculosis: A cross-sectional analysis
Source: PLOS Glob Public Health. 2024 Feb 8;4(2):e0002892. doi: 10.1371/journal.pgph.0002892 (PMC10852290; doi:10.1371/journal.pgph.0002892)
Supplement: Attachment — Submitted filename: 7.5 submission response to reviewers.docx [file pgph.0002892.s004.docx]

8/22/2023

To whom it may concern:

Thank you for your feedback. I have updated the manuscript to address the items below as outlined in your email.

1. Your study includes live participants, but you have not included an Ethics Statement. Please include an Ethics Statement subsection to your Materials and Methods, to include:

i) The name(s) of the Institutional Review Board(s) or Ethics Committee(s)

ii) The approval number(s), or a statement that approval was granted by the named board(s)

*“Regulatory approval was obtained from the Makerere University School of Medicine Research and Ethics Committee of the College of Health Sciences (Ref: MakSOMREC 2021–54 and the Virginia Commonwealth University IRB (HM20020265)”* was added to the methods section.

iii) (for human participants or donors) - A statement that formal consent was obtained (must state whether verbal/written) OR the reason consent was not obtained (e.g., anonymity).

*“All subjects who completed questionnaires by phone provided verbal witnessed consent via phone in accordance with local regulations regarding human subject interviews.”* Was added to the methods section.

2. Please complete all items on the Clinical Studies Checklist that are relevant for your submission, by following this link: http://journals.plos.org/plosone/s/file?id=dc11/PLOSOne_Clinical_Studies_Checklist.docx.

The Clinical Studies Checklist, ethics committee approval, and STROBE checklist were uploaded as “other” to the submission.

Please do not hesitate to reach out if you need any more information. Thank you for your consideration.

Sophie Wennemann

VCU School of Medicine

Class of 2025
