# Supplementary material for: The effect of biomass smoke exposure on quality-of-life among Ugandan patients treated for tuberculosis: A cross-sectional analysis
Source: PLOS Glob Public Health. 2024 Feb 8;4(2):e0002892. doi: 10.1371/journal.pgph.0002892 (PMC10852290; doi:10.1371/journal.pgph.0002892)
Supplement: Attachment — Submitted filename: BiomassSGRQ_ReviewerLetter_final.docx [file pgph.0002892.s005.docx]

Journal Requirements:

1.In the ethics statement in the Methods, you have specified that verbal consent was obtained. Please provide additional details regarding how this consent was documented and witnessed, and state whether this was approved by the IRB"

*A statement addressing this point was added into the first paragraph of the Methods section*

2. We have noticed that you have uploaded Supporting Information files, but you have not included a list of legends. Please add a full list of legends for your Supporting Information files after the references list.

*A list of legends was created and uploaded*

**Additional Editor Comments (if provided)**:
Please, respond to all the reviewers' comments particularly, reviewer#3. In addition to reviewer#3 comments on exposure definition, note that the way in which exposure is defined will introduce serious exposure misclassification. (see Maggie L. Clark et al 2010:Indoor air pollution, cookstove quality, and housing characteristics in two Honduran communities. Environmental Research 110 (2010) 12–18) and needs to be discussed

*Thank you for this comment. Information regarding stove type, fuel type, and separate rooms/ventilation was collected for these subjects. However, based off of the data we had collected, the objectives of the study, and the small numbers of subjects within each group when separated by fuel type, ventilation, and stove type (which would result in diminished power), we present years exposed here and acknowledge the need for further study.*

Reviewers' comments:

Reviewer #1: The article meets a recognized need of patient related data on quality of life of Uganda patients with TB exposure and treatment who might have concomitant exposure to indoor air pollution from biomass fuel. Unfortunately, in its current state it partially fails to meet the criteria for PLOS Global public health journal publication and has a flawed manuscript with conclusions that are supported by a seemingly compromised data collection process. The cross sectional study design for the primary research question posed projects an ethically and methodologically sound design with limitations and weaknesses that can be fully analyzed and presented. However, In my opinion, data collection process is a science which needs mastery execution to validate the data collected.
An attempt at statistical analysis of the data is seen through the mathematical formulas to infer or defer any data correlation or association but its meaning remain tainted with the aforementioned shortcomings. Additionally, a comment on data availability cannot be made at this point as the reviewer failed to access the supplementary data in its .xlx format.
In attempt to revise the aforementioned shortcomings, the following recommendations can be considered.
*Thank you for your comments and recognizing the importance of exploring this relationship between quality of life, biomass expousure and TB infection. We are happy to provide the data set again if needed, we did upload this previously. In regards to our data selection we hope that our responses below will address your concerns.*

Line 32- the use of three data collection tools is commendable particularly in consideration of the type and quality of data that needs to be gathered to answer the posed question, however, a critical analysis of the data collection tools themselves needs to be provided in the form of a rationale for why they were selected and their advantages and disadvantages to the task they are being set to accomplish e.g how the tool depends on recall bias, language and understanding etc.

*Thank you for this comment, further information on why the data collection tools were chosen was added to paragraph 3 of the Methods section with relevant citations to support their validity in Uganda and in TB research*

Line 63- author’s access to unblinded data is mentioned but is not acknowledged as a weakness in the limitations section. This mention as a weakness is validated by the potential author report bias that could affect the data collection and synthesis process. Additionally, a detailed mentioning of which authors had access to the information would strengthen evidence based on a critical analysis of the data collectors skill/qualification to do so.
*Thank you for pointing this out. More information about this was added to the first paragraph of the methods section. The authors did not have access to identified data during analysis, we apologize for the confusing language previously.*

Line 72 and 92- there is paucity of information on the high attrition rate of 46% eg why 46% did not complete the interview and what was or could be done to reduce this high rate.

*Thank you for this comment. The final numbers and exclusions have been clarified in the Methods section, and a consort diagram has been created (Figure 1). There was not an attrition rate of 46% I believe this is in reference to the number of individuals with phone numbers in the medical chart but does not reflect those who were actually able to be reached for interview.*

Line 74- provide an adequate description and rationale of the randomization process to select the 1/4 selected to complete questionnaires AND detail on the implementation of the phone interviewing eg call duration, language used, data collector’s qualifications etc because this helps judge the quality of evidence collected especially considering how a ‘visual’ analog scale was conducted through a phone interview(line80).

*Thank you for this comment, more information about the language used and data collector’s qualifications were added to the first paragraph of the methods section. Unfortunately, we do not have exact information on call duration but the subjects in this study had numerous questionnaires to complete and the decision was made to limit the number who had QoL questionnaires to ¼ subjects. Every 4^th^ subject called and consented for the interview was selected to complete these surveys, subjects were called at random from selected charts in our TB clinics. This sampling procedure is detailed in our paper* PLOS Global Public Health | <https://doi.org/10.1371/journal.pgph.0001573>. I*nformation was added into the second paragraph of the Methods section to more thoroughly explain the sampling procedure. Additionally, clarification that the subjects completed the EQVAS verbally was added to the third paragraph of the methods section. The EQVAS is commonly completed verbally in the case of phone interviews and EuroQol has interviewer-administered versions available for such cases, citations were added here as well for context.*

Line 95- provide rationale why the selected confounders were selected from a very wide range of confounders that could potentially misrepresent the meaning of the collected data. eg the recognized and mentioned limitations that lack of spirometric data, pre-existing pulmonary function etc. could also confound the result’s interpretation. in the same vein, the ‘chart review’ process for data collection on cardiopulmonary comorbidities was no fully described and might need clarification on which chart was reviewed and if it was the best one to use for such relevant data seeing as this would affect interpretation of the results.

*Information about why the confounders tested for were chosen was added to paragraph 5 of methods. The chart review process and how it was confirmed with patients via interview was also further described in this paragraph.*

Line 104- There is an obvious discrepancy on the total patients who completed each questionnaire and the existence of this difference is not explained. This might challenge the comparability of the tools used for data collection (line 148) if not challenging the accuracy and validity of the actual result concluded for each tool.

*Thank you for this comment, while all subjects selected to complete QOL questionnaires were asked to complete all three instruments a small number of subjects ended calls or calls were dropped due to lost signal during the interview which did not allow all questionnaires to be completed. However, all complete surveys were included in their respective analysis. We have clarified this in the consort diagram legend (Figure 1). We have also added a line addressing this as a possible source of selection bias in our discussion section.*

Reviewer #2: Rationale
• It is not clear why study was done among TB patients. QoL due to biomass fuel can be poor irrespective of the TB status.

*While biomass fuel exposure can worsen QOL we were particularly interested in the interplay of QOL after TB infection in those with biomass smoke exposure as a potential risk factor for ongoing pulmonary symptoms and suboptimal treatment outcome for TB. There is growing interest in post-TB lung disease, which is defined as respiratory symptoms and pulmonary dysfunction after microbiologic cure. While this study is not definitive it provides insight into the potential contribution of biomass smoke exposure as a risk factor for worse QOL after TB treatment. This information was added to the discussion section to make the connection more clear.*

• Is the objective descriptive or analytical?

*This was an analytical study performed to assess the association between length of biomass smoke exposure and perceived quality of life of post-cure TB patients in Uganda*.

Methods
• The rationale for light/heavy exposure cut-offs not clear

*This was added to paragraph 3 of methods. Heavy exposure correlated to the top quintile of our cohort.*

• What was the objective for which sample size was calculated?
• What were the assumptions for sample size calculation?
• Was sample size adequate for analytical study?

*Thank you for bringing up these questions. The following is a secondary analysis of a study aiming to examine characteristics of patients treated for TB during the COVID19 pandemic in Uganda. As such, there were no a priori sample size estimates for this analysis. The present results are not intended to be representative of the Ugandan population. A study of that nature would require random sampling of health centers across all rural and urban areas in the country, however, as specified we did attempt to collect data from a representative group of urban and rural clinics that were both primary and tertiary TB care delivery sites. We have clarified this in the discussion. Although no a priori sample calculations were performed, a conservative estimate based on data from Katoto et al (*doi: 10.3390/ijerph15112563) *suggests that a sample of 146 would be sufficient to detect an OR of 2.7 assuming with power of 80% and type 1 error of 0.05, to detect worse symptoms in subjects previously treated for TB with biomass exposure.*

Results
• The first para should describe QoL as per various scales. Objectives seem descriptive, but the results seem like an analytical study.

*As stated above, this was an analytical study performed to assess the association between length of biomass smoke exposure and perceived quality of life of post-cure TB patients in Uganda. The purpose of the various QOL questionnaires and their utility is addressed in previous comments. If the reviewer would like the first paragraph to have more detail on QOL questionnaires we are happy to reformat to include details in that section.*

• Whether the biomass fuel exposure was current or in the past? With access to clean fuels, people might have changed their cooking fuel in the recent years.

*Data was collected on both previous biomass exposure without ongoing exposure and current exposure. We have clarified this in the beginning of the results section and further addressed it in the discussion.*

• Describe the data as per various domains

*We apologize, we are not clear what the reviewer is referring to. Please let us know which domains or analysis you would like us to further explain.*

• Too many models with no clear message. It is not clear if sample size is sufficient for such analysis.

*We performed a basic association between biomass exposure and QoL in a group of TB patients via phone delivered interviews while controlling for basic confounders. We have added information about our sample size analysis in the methods session and addressed it in questions above.*

Discussion
• If the objective was to see whether TB patients with biomass exposure had worse outcomes, a comparison group of non-TB patients should have been included in the study design. In the absence of control group, no interpretation can be made whether QoL is any different for TB patients.

*Thank you for this comment. This was mentioned in the discussion section as a limitation of the study. The objective of interest was clarified in the discussion to be more specific to TB patients in regards to post-TB lung disease rather than just QOL in previous TB patients vs. those without previous TB. A comparison of non-TB patients with biomass exposure would not account for TB as a risk factor for lower respiratory QOL which has been shown to be true.*

• Discussion should focus on how the QoL compared with other TB studies in the same country/ similar settings.

*Thank you for pointing out the lack of this. While there are very few studies investigating this association in this population, a paragraph was added to the discussion section with what is available.*

Reviewer #3: 1. Concern about study design :
The study design is announced in the the discussion section (Lines 145, 157). Isn't it better to specify this at an earlier stage, to fix the reader's mind?
Suggested places : abstract, methodology and eventually in the title.

*Thank you for this comment. I have added this information in the title and introduction as well.*

2. Concerns about samples and discrepancies between some sections

a. What population is studied ?
The authors say: « Beginning in February of 2022, Medical records were reviewed for patients receiving TB care from September, 2019 to September, 2020 at three urban and three rural TB clinics in Uganda ». lines 65 to 67
The first idea that emerge from this paragraph is that the studied group is TB patients (i.e. currently undergoing treatment). However, the dates seem to point towards previous patients. If Uganda applies the same treatment durations as we know (6 or 12 months), the cohorts from September 2019 to September 2020 are expected to complete their treatment before data collection in February 2022. Your discussion seems to support my point of view when you write "... previously treated for TB (L145-146)...". However, immediately afterwards, you refer again to patients with TB L146-148...". Isn't TB curable? So people who have been treated and declared cured from TB are no longer patients. In my opinion, these two concepts cannot be used for each other, as they refer to very different study populations.
Suggestions: it's important to make a choice and maintain consistency by talking about either "TB patients" or " previous patients" and not both.

*Thank you for pointing out this important discrepancy to us. The study was looking at previous TB patients. We have gone through the paper and ensured that this is corrected.*

b. sample representativeness

The authors state that subjects were selected from 6 structures (3 urban and 3 rural). However, the authors do not say anything about the number of medical centers that care for TB patients in Uganda, or about the selection technique used to select these 6 structures (whether random or not). At the same time, the title, objective and conclusion seem to suggest that the study results are applicable to Ugandan TB patients (or previous TB patients).
Questions: Is the sample of TB patients drawn from these 6 medical centers representative of Ugandan TB patients? The secondary questions to which we would like to find answers in the methodology are: Are the 6 selected facilities representative of the facilities that care for TB patients in Uganda? What weight do the patients cared for in these 6 facilities represent on a national scale? What is the spatial distribution of these 6 facilities across Uganda?
Suggestion: It may be necessary to provide more details on sampling or to adapt the title, objective and conclusion.

*The 6 medical centers that were selected across the country were chosen as an effort to obtain a representative sample of the population of TB patients in Uganda. These hospitals represent some of the largest referral hospitals as well as smaller referral hospitals in different geographic locations throughout Uganda.*

c. Sample size
How was the sample size estimated ? If exhaustive sample, please specify it.
Line 72 « Of these individuals, 54% (710/1320) and 672 completed interviews ». Is this sentence complete ? Finally, how much completed interviews :710 or 672 ?
Suggestion : It may be useful to add a flow chart showing the steps used to select the subjects included in the study and the stratification of respondents into the 3 groups.
*Thank you for this comment, this error was corrected and a consort diagram was created, see previous comments on sample size above*

3. Exposure concerns
Curiosity: what exactly does "zero exposure" mean? We know that there are many places of exposure, and that people can be passively exposed, for example in transport, in public places, during bush fires, ..... I don't personally believe that there are people with zero exposure. But if a threshold value has been used, if certain exposures have been minimized, this should be clearly indicated.

*Thank you for this comment, I have changed the wording throughout the paper to “no-reported exposure” instead of “no exposure” and explained the use of this term in the methods section.*

Exposure to smoke was investigated by starting from the interview period and working backwards, but the backward period was not defined in the paper. How was recall bias controlled? Similarly, the way in which years are counted needs to be clarified in the methodology, given that there may be periods of non-exposure due to travel, for example.

*Recall bias is a concern in this study when asking about biomass smoke exposure, a sentence about this was added in the paragraph outlining limitations in the discussion section. Patients were asked how many years they have lived in a home that uses biomass fuels for cooking/home heating/etc. both currently and in the past if applicable. We have also changed the wording describing this in the methods section so it is clearer how the information was collected.*

4. Results presentation
Is it not important to present some descriptive and inferential statistics in the tables?

*Thank you for bringing up this important point, 2 tables were created for this purpose.*

Reviewer #4: Wenneman et al, in their paper “The effect of Biomass Smoke Exposure on Quality-Of-Life among Ugandan Patients treated for Tuberculosis” aimed to investigate the association between biomass smoke exposure and self-reported quality of life scores in a cohort of TB patient in Uganda. For this study, participants had been taken from the sample of a cohort study in Uganda on the effect of biomass smoke exposure on the risk of TB diagnosis. Here, researchers reviewed medical records of patients who were taking TB care from September, 2019 to September, 2020 from three unban and three rural TB clinics in Uganda. With verbal consent from the participants over phone, researchers randomly selected participants to complete three validated quality-of-life surveys including the St. Georges Respiratory Questionnaire (SGRQ), the EuroQol 5 Dimension Level system (EQ-5D-3L) and Patient Health Questionnaire 9 (PHQ-9). They also compared the self-reported quality-of-life of the TB patients with the exposure to biomass smoke that categorized into three groups depending on years of smoke exposure – no exposure (0-years), light exposure (1-19 years), and heavy exposure (20+ years). In aiming to obtain the result, analyzers performed independent-samples-Kruskal-Wallis testing with post-hoc pairwise comparison and the Bonferroni correction. In this paper they revealed significant relationship of the quality-of-life of TB patients on treatment with exposure of biomass smoke. After critical analysis of the findings of the survey and bio-statistical analysis, they were able to discover the relationship of biomass smoke exposure and quality of life of TB patients.

The strength of this paper is that it addressed an interesting and contemporary question, and was able to identify the pessimistic impacts of biomass smoke on the quality of life of TB patients. This article also inspired us to think about the adverse consequences of other factors on health outcomes of TB patients even those are on anti-Tubercular medications. This study also clearly demonstrated its limitations and suggested for further evaluation through another similar studies.

However, this study includes some deficiencies. One of the weaknesses of this article is occasional incomprehensiveness which establishes unclear logical links between concepts. In addition to this, method of sampling seems to be little confusing, particularly in line no 72. Another possible weakness could be the discussion part which might be more descriptive and topic oriented.

In spite of having some weaknesses of this article, it is published worthy as it has added a new concept in the field of research.

*Thank you for this assessment of our study’s strengths and weakness. We have clarified the sampling process (see above comments) and significantly expanded the discussion section to include connections to other relevant literature and clarification about our conclusions drawn from this study.*
