# Supplementary material for: The effect of biomass smoke exposure on quality-of-life among Ugandan patients treated for tuberculosis: A cross-sectional analysis
Source: PLOS Glob Public Health. 2024 Feb 8;4(2):e0002892. doi: 10.1371/journal.pgph.0002892 (PMC10852290; doi:10.1371/journal.pgph.0002892)
Supplement: Attachment — Submitted filename: Response to Reviewers 1.11.24.docx [file pgph.0002892.s006.docx]

Response to Reviewers

January 11, 2023

Reviewer #1: A significant improvement from the original submission. The revision addressed proposed recommendations and remains with a few areas for more improvement as pointed out below.

Line 99- “ although no a priori sample calculations were performed “ possible grammatical error.

*Thank you for pointing this out. The wording was changed to “although a priori sample calculations were not performed” to avoid grammatical errors.*

Line 111- grammatical error, The EQ-5q-3l

*Thank you, this has been corrected.*

Line 140- Give the meaning of the acronym IQR at the bottom of the table like what is done for SES.

*Thank you, I have added this clarification under the table and in the supporting information section.*

line 173- The table format is not showing the last column after standard error (on the reviewer’s screen it appears just cut off from the end of the page).

*I have double checked that the tables are uploaded into the document as instructed by the PLOS Global Public Health submission guidelines. If the reviewer is still unable to see the tables when the document is in draft mode, please let us know and we would be happy to upload them separately.*

Line 166- The kruskal-wallis one way analysis of variance results are missing the number before the decimal point and your reader may not know this is automatically a zero so a recommendation would be to be uniform.

*Thank you for bringing this to light. I have gone through each file and ensured this is uniform as suggested.*
